# Strategies for improving cathode electrolyte interphase in high-performance dual-ion batteries

**DOI:** 10.1016/j.isci.2024.110491

**Published:** 2024-07-11

**Authors:** Yitao He, Zhipeng Chen, Yaohui Zhang

**Affiliations:** 1 Department of New Energy Science and Engineering, School of Energy and Environment, Anhui University of Technology, Ma’anshan, Anhui, China; 2 School of Physics, Harbin Institute of Technology, No. 92 Xidazhi Street, Harbin, Heilongjiang 150001, China; 3 Department of Thin Films and Nanostructures, FZU – Institute of Physics of the Czech Academy of Sciences, Cukrovarnická 10/112, 162 00 Prague 6, Czech Republic

**Keywords:** electrochemistry, energy application, energy materials

## Abstract

Dual-ion batteries (DIBs) offer high energy density due to the ability to intercalate both anions and cations, thereby increasing the cutoff voltage and battery capacity. Graphite, with its ordered layered structure and cost-effectiveness, is commonly employed as the cathode material for DIBs. However, the discharge capacity of graphite cathodes is relatively low, and their cycling stability is poor, limiting the practical applications of DIBs. The formation of cathode electrolyte interphase (CEI) on the graphite cathode surface is closely related to anion behavior. Constructing a stable cathode electrolyte interface is crucial for improving the stability of anion storage. Therefore, we introduce a series of strategies to enhance the quality of the CEI layer, including additives, binders, main salts or solvents, high-concentration electrolytes, doping elements, artificial CEI, and graphite surface modifications. These strategies improve the CEI by enhancing anion transport rates, increasing anion solvation capabilities, and improving the structural stability of graphite cathodes, which is of profound significance for increasing the capacity and stability of DIBs. This review provides inspiration for future CEI research, encouraging further exploration of resources of CEI components and improvement strategies to further promote the development of DIBs technology.

## Graphical abstract

**Figure:**
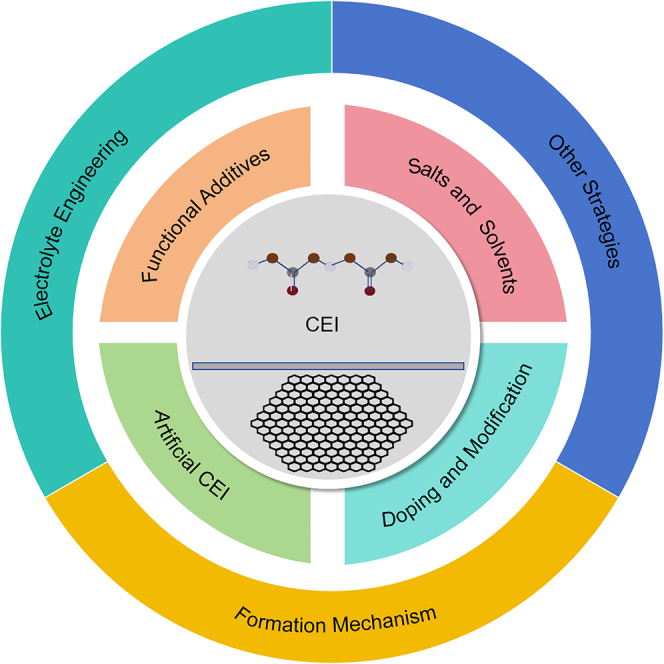

## Introduction

In recent years, lithium-ion batteries (LIBs) based on cation intercalation have been driving society’s energy demands.^1^^,^^2^^,^^3^ Traditional LIBs mainly focus on the intercalation and de-intercalation processes of lithium ions between positive and negative electrode materials, with relatively less attention given to the utilization of anions in the electrolyte.^4^ As early as 1938, Rüdorff and Hofmann discovered that anions could be intercalated into graphite through chemical or electrochemical means, and were considered as a potential positive electrode material for batteries.^5^ However, traditional anion intercalation typically requires the use of highly concentrated acid solutions as electrolytes, which poses a series of safety issues.^6^ In the 1990s, Carlin et al.^7^ reported the use of room temperature ionic liquids as electrolytes in a dual-graphite intercalation molten electrolyte battery, successfully demonstrating the application of anion-intercalated graphite as a positive electrode material. Over the following decades, progress was made in anion-intercalated graphite-based dual-carbon batteries (DGIBs), including achieving anion intercalation in non-aqueous electrolytes, *in-situ* characterization of stage-wise anion intercalation processes, and systematic studies on the intercalation of different anions into graphite.^8^^,^^9^

Recent studies have indicated that in DIBs, the potential for anion intercalation is higher than that for Li/Li^+^,^10^^,^^11^ which may lead to electrolyte decomposition and solvent molecule co-intercalation. In the state of co-intercalation, some anions may remain trapped within the graphite layers, inducing the irreversible distortion of the graphite crystal structure, thereby leading to low Coulombic efficiency and poor stability. Moreover, the interlayer spacing of graphite is 0.335 nm,^12^ much smaller than the diameter of intercalated anions. Therefore, after prolonged cycling, the layered structure may be disrupted, leading to capacity degradation. Generally, a stable and uniform CEI effectively isolates the cathode from the electrolyte, hindering the kinetics of side reactions, thereby allowing reversible electrochemical reactions to operate stably over the long term. Researches have shown that a stable CEI is crucial for enhancing electrode kinetics and improving battery performance.^13^^,^^14^^,^^15^ Competitive electrochemical oxidation reactions between solvents and anions occur on the cathode surface, forming a layer of an insoluble product known as CEI.^16^^,^^17^ The thickness of the CEI layer theoretically ranges from a few nanometers to tens of nanometers, largely depending on the properties of the electrolyte solution and the cathode material.^18^^,^^19^ A stable CEI should possess high ionic conductivity and low electronic conductivity while maintaining a stable body structure and chemical stability to suppress cathode material separation or cracking during volume changes.^20^^,^^21^

However, disappointingly, spontaneously formed CEI is typically uneven and unstable. Although DIBs have a high charge cut-off voltage, allowing for higher energy density, it can lead to the oxidative decomposition of the electrolyte, producing gases and unwanted byproducts.^22^^,^^23^^,^^24^ Anions attempting to migrate out of the graphite layers inevitably cause distortion to the crystal structure of the graphite on the cathode surface,^12^ resulting in the uneven oxidation of the electrolyte and the formation of CEI. Additionally, the phenomenon of solvent molecule co-intercalation in the cathode material can also lead to irreversible changes in the graphite crystal structure, resulting in the unstable growth of CEI.^10^^,^^11^ Furthermore, the dynamic formation and dissolution of CEI during charging and discharging processes can also affect the stability of the CEI structure.^19^^,^^25^ The electronically insulating CEI layer can also increase the resistance between the electrode and the interface, potentially leading to power losses and inhibiting the kinetics of ions.^26^ In DIBs, the CEI plays a crucial role. It is a solid layer formed on the electrode surface, composed of decomposition products of the electrolyte and oxides of the electrode material. It is in direct contact with the electrolyte and influences the anion transfer efficiency between the electrode and the electrolyte, which has a significant impact on the performance and safety of the battery. The formation of the CEI can prevent adverse reactions between the electrode material and the electrolyte, enhancing the battery’s cycling stability and safety. Furthermore, the CEI can reduce the internal resistance of the battery, improve charge/discharge efficiency, thereby enhancing the battery’s energy density and power density. Therefore, a deep understanding of the resources of CEI components is crucial for determining the most stable CEI structure, thus determining the performance of longer cycle batteries. This review focuses on the composition of the CEI on the graphite cathode in DIBs and discusses its impact on battery performance.

The formation mechanism of CEI is that during the charging and discharging process of DIBs, the solvents and salts in the electrolyte will be affected by the electrochemical reaction on the surface of the electrodes when the redox reaction occurs and decompose to produce various chemical substances. Among them, some decomposition products will form a thin CEI on the electrode surface. CEI has many advantages in DIBs. Firstly, a uniform and dense CEI can make the current distribution on the electrode surface more uniform. When the current is uniformly distributed on the electrode surface, local overpotential differences caused by uneven current density can be avoided, thus reducing the problem of inconsistent local electrochemical reaction rates. This helps to improve the working efficiency and performance of the electrode, and reduces local corrosion and damage on the electrode surface, reducing the degradation rate of the electrode and thus extending the service life of the battery. Second, high-quality, high-density, low porosity CEI can effectively isolate side reactions between the electrodes and electroactive substances and the electrolyte. The side reactions may lead to loss of battery capacity, reduction of electrochemical efficiency, and destruction of electrode materials, and the occurrence of these side reactions can be reduced by forming excellent CEI, improving the stability and reliability of DIBs. In addition, CEI improves the desolvation effect. In electrolytes, ions usually exist surrounded by solvents. These solvent molecules must be stripped off to enable ions to pass through the graphite cathode surface when the anions intercalates. By constructing a CEI on the graphite cathode surface, it can help to achieve this desolvation process and further improve the ion transfer rate of the battery. Therefore, in order to achieve the high performance of DIBs, it is essential to construct a suitable CEI on the graphite cathode surface.

This review first introduces the resources of CEI components, which is a complex process involving the reaction of solutes, electrolyte decomposition, and ion migration, among others. On this basis, various optimization strategies for the structure and composition of CEI are comprehensively discussed. Regarding the structure and composition of CEI, optimization can be achieved through material synthesis, additive regulation, and other means. For example, the introduction of additives can enhance the stability of CEI, improve ion transfer, and mitigate battery polarization issues. Later, the research process and characterization methods of the CEI are introduced. Characterization methods include electrochemical tests, surface analysis techniques, such as scanning electron microscopy (SEM), transmission electron microscopy (TEM), X-ray photoelectron spectroscopy (XPS), and Raman spectroscopy. Finally, an outlook is provided for the development of a more stable CEI. With the rapid development of DIBs, the requirements for the stability and performance of CEI are constantly increasing. Therefore, further research on the formation mechanism, optimization strategies, and characterization methods of CEI, as well as the development of new materials and technologies, will help achieve a more stable and efficient CEI, and further promote the application of DIBs. This review provides a systematic reference for the development of CEI and guidance for further research and optimization of CEI formation and performance.

## Resources of cathode electrolyte interphase components

In DIBs, the resources of CEI components are one of the crucial factors affecting battery performance. During the discharge and charge processes of DIBs, the anionic solvation sheath in the electrolyte adsorbs on the graphite cathode surface and partially enters into the graphite layers, leading to reactions between certain components in the electrolyte and the graphite. These reactions result in the formation of a CEI layer, which further covers the surface of the graphite particles and interacts with the components in the electrolyte. This CEI layer plays a vital role in battery performance as it regulates the transport rate of anions, while protecting the graphite from further electrolyte decomposition and structural degradation. Therefore, understanding the resources of CEI components in graphite is crucial for optimizing battery performance and enhancing cycling stability.

**Figure 1:**
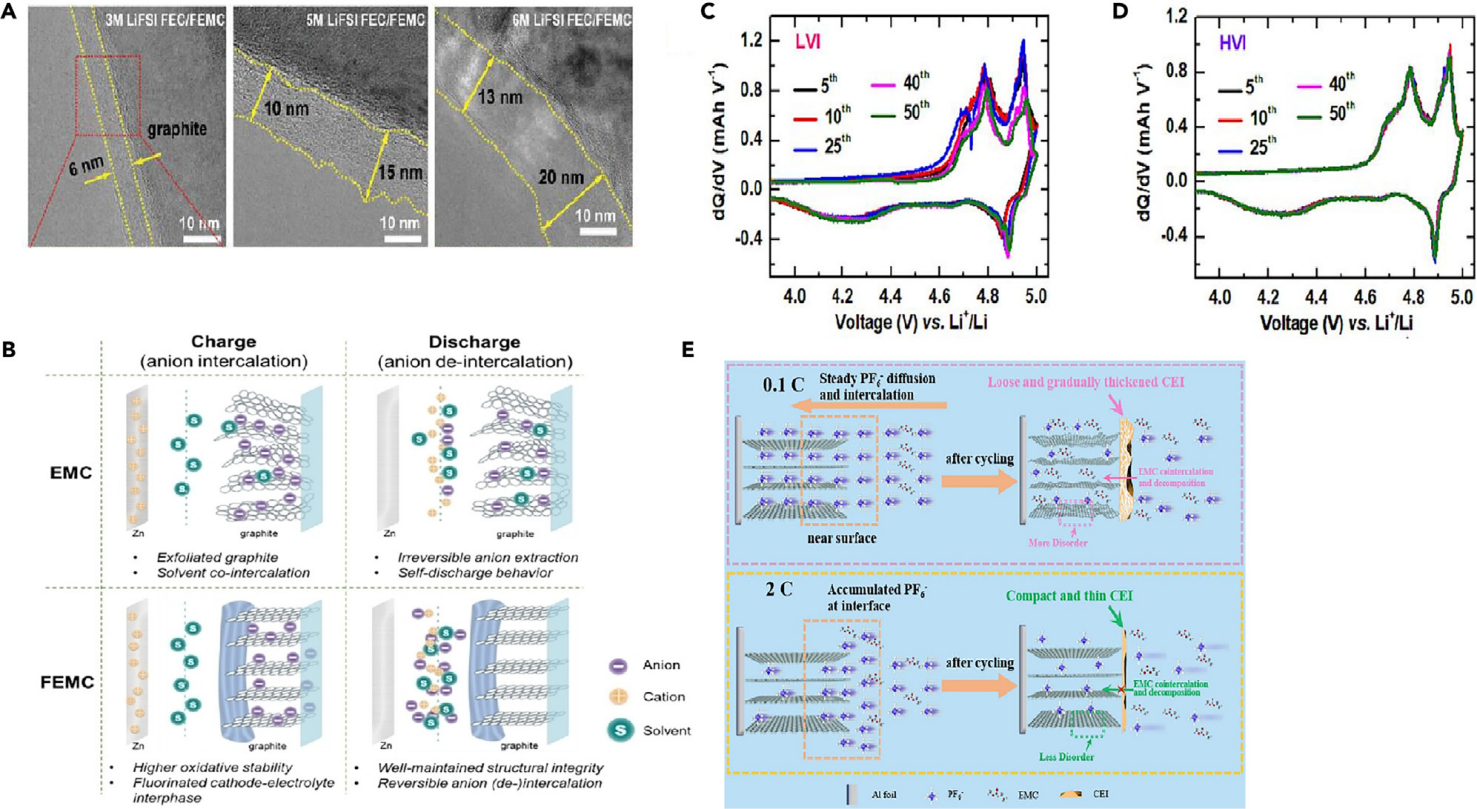
TEM of SEI, and schemes of reactions (A) High-resolution TEM images of graphite cathode in 3, 5, 6 M electrolytes (LiFSI FEC/FEMC = 3:7). Reprinted with permission from Zhao et al.^30^ Copyright 2023 Wiley-VCH GmbH. (B) Schematic illustration of the proposed mechanisms for a Zn-graphite EMC/FEMC cell during charge (anion intercalation) and discharge (anion de-intercalation). Reprinted with permission from Tao et al.^31^ Copyright 2023 Wiley-VCH GmbH. (C and D) Differential capacity profiles of 5th, 10th, 25th, 40th, and 50th cycles during the anion-storing performance – (C) LVI, and (D) HVI. Reprinted with permission from Ghosh et al.^32^ Copyright 2023 Elsevier Ltd. (E) Schematic illustration of the differences in electrolyte reactions, CEI and graphite structures after cycling at 0.1C and 2C. Reprinted with permission from Jiang et al.^33^ Copyright 2024 Elsevier Ltd.

It was found that the composition of CEI mainly comes from the combined action of salt and solvent molecules in the electrolyte, and is a product of the redox reaction of anion solvation behavior on the anode surface. In order to reveal the fluoroethylene carbonate (FEC)-regulated anion solvation behavior, Yu et al.^27^ systematically investigated the structural changes of graphite cathode during the cycling process as well as the differences of the electrolyte/cathode interfaces in two different electrolytes by various physical means, and proved that the composition of the CEI layer originated from the solvent molecules and salts in the electrolytes. Wang et al.^28^ analyzed the morphology and chemical composition of the graphite electrode surface after ten charge/discharge cycles using TEM and XPS. TEM images revealed the presence of amorphous and small layers sporadically adhering to the graphite particles. The formed CEI was irregular and non-uniform. Due to the high cut-off voltage of 5.2 V, organic components in the CEI can decompose under this high voltage, which may further contribute to the formation of LiF. This can somewhat impact the morphology and structure of CEI. Additionally, these irregular layers were primarily scattered on the basal planes of the graphite particles. The C-C, C-O, C=O, ROCO_2_Li, and LiCO_3_ peaks were observed in the C1s spectra of all these graphite electrodes, originated from the solvent decomposition on the graphite cathode surface. Furthermore, F1s spectra indicated the presence of LiF, Li_x_PO_y_F_z_, and PF_6_^−^. The generation of LiF and Li_x_PO_y_F_z_ can be attributed to the decomposition of LiPF_6_, while the presence of PF_6_^−^ indicates the retention of PF_6_^−^ anions in the CEI layer, leading to irreversible capacity loss in DIBs. Wang et al.^29^ suggested that by constructing a robust and favorable surface layer, the decomposition of the electrolyte and structural breakdown of the graphite cathode can be suppressed, thereby optimizing PF_6_^−^ intercalation channels and enhancing cycling performance. They believed that the differences in electrochemical performance may stem from the variations in the CEI layer, formed as a result of reactions with the electrolyte. Zhao et al.^30^ achieved a stable and thin CEI layer by adjusting the solvation structure of the electrolyte and creating a favorable electrolyte environment. Simply increasing the concentration of LiFSI salt does not necessarily improve battery performance. Higher salt concentrations lead to decreased ion conductivity, slower ion migration rates, and greater side reactions on the graphite surface, resulting in an increase in CEI layer thickness. The concentration of salt and solvent should be coordinated to form a uniform and stable CEI layer to enhance performance (Figure 1A).

Tao et al.^31^ described a comparison between the ethyl methyl carbonate (EMC) and methyl 2,2,2-trifluoroethyl carbonate (FEMC) systems in the development of a schematic diagram (Figure 1B). The key mechanistic insights from the diagram are summarized, focusing on the observed phenomena during the charging (anion intercalation) process in the EMC-based electrolyte. During charging, it was observed through various experimental techniques, such as Raman spectroscopy, XRD, SEM, and HRTEM, that graphite exfoliation and interlayer overexpansion occur. The main cause of these phenomena was identified to be the interaction and co-intercalation of the EMC solvent with graphite, which was validated through the accumulation of species containing C=O, C-OH, and C-*O*-C bonds, as well as calculated binding energies and molecular dynamics simulations. The interactions between the solvent and the cathode in the EMC system hinders and impedes the intercalation and de-intercalation of anions. As a result, during the discharge process, the inserted anions cannot be fully extracted, leading to irreversible anion transport and loss. Repeated charging and discharging cause the accumulation of a significant number of non-extractable anions within the graphite interlayers, resulting in a permanent reduction in capacity and a severe decline in battery performance. Additionally, besides the oxidation decomposition of the electrolyte, significant self-discharge behavior was observed. In contrast, the FEMC based electrolyte effectively suppresses self-discharge behavior. In addition to the inherent higher oxidation stability of FEMC, this suppression can be attributed to the formation of a fluorinated CEI layer, which helps maintain the structural integrity of the graphite surface and interlayers. Consequently, a more stable and reversible anion (de-)intercalation process was achieved, leading to an overall improvement in battery performance.

The composition and structure of the CEI layer formed in DIBs undergo changes during battery operation − a dynamic process involving an increase or decrease in CEI layer thickness and changes in the layered structure. Ghosh et al.^32^ compared the performance of two DIBs interfaces: the high voltage interface (HVI) and the low voltage interface (LVI) under a fluorine-containing environment. They found that more interface side reactions occur at the LVI compared to the HVI. This was due to the structural, organic, and inorganic compositional layering as well as the reactivity of the fluorine-containing environment. While LiF content is higher in the LVI, it predominantly resides in the inner layer, whereas the outer layer of the LVI, in direct contact with the electrolyte, is rich in polycarbonate and polyether, which may undergo severe side reactions and irreversible charge consumption. In contrast, the relatively stable fluorinated organic dimer might form an outer covering, minimizing interface side reactions to the greatest extent and restricting CEI growth under HVI conditions. Furthermore, the continuous evolution of organics-rich CEI throughout the cycling life leads to poorer electrochemical performance in the LVI. The dynamic evolution of CEI can be traced from the differential capacity profiles (Figures 1C and 1D). The initial cyclic capacity spectra of the HVI overlap, indicating minimal changes in CEI once formed. In the LVI, CEI continues to undergo random changes even after its formation, as observed from the non-overlapping nature of the differential capacity spectra. Therefore, the interface stability brings superior electrochemical performance to the HVI.

Jiang et al.^33^ investigated the influence of different current rates on the reaction of PF_6_^−^ ions, the formation of the CEI, and the crystalline structure of graphite (Figure 1E). At a relatively low current rate of 0.1 C, PF_6_^−^ ions diffused and intercalated steadily into the graphite layers during the initial charging period. Near the surface region of the graphite, the concentration of PF_6_^−^ anions was not high because they had sufficient time to intercalate into the graphite at low current densities. Consequently, the CEI formed from the initial decomposition of LiPF_6_ was relatively loose, unable to effectively prevent electrolyte co-intercalation and decomposition in subsequent cycles. Therefore, the CEI layer continued to grow and thicken. Additionally, the low charge rate favored more complete electrochemical reactions, leading to solvent EMC reacting with the graphite lattice, ultimately disrupting the layered structure of graphite and generating a large amount of disordered carbon. Conversely, at a high charge rate of 2 C, due to limited diffusion kinetics within the graphite layers, PF_6_^−^ ions might accumulate on the surface of the graphite. Thus, the CEI layer formed from the decomposition of LiPF_6_ was dense and thin, significantly reducing electrolyte co-intercalation and decomposition reactions on the graphite. The crystalline structure of the graphite was better preserved under these conditions consequently.

Most additives are used as resources containing highly reactive LiPF_6_ salts to form CEI.^34^ The partial decomposition of LiPF_6_ is a well-documented phenomenon with the following reaction process:

(Equation 1) $${\text{LiPF}}_{6}\to \text{LiF}+{\text{PF}}_{5}$$

Hydrolysis of PF_5_ leads to the formation of HF. It is well known that the generated HF reacts with the surface impurities of various cathode materials (e.g., LiNi_0.8_Co_0.2_O_2_),^34^ leading to further accumulation of LiF:

(Equation 2) $${\text{Li}}_{2}{\text{CO}}_{3}+2\text{HF}\to 2\text{LiF}+H_{2}O+{\text{CO}}_{2}$$

The composition of the CEI obtained in LiPF_6_-rich electrolytes is influenced by a number of factors, including cutoff potential,^35^ operating temperature,^36^ cathode material, and electrolyte formulation. Typically, the CEI layer formed will contain significant amounts of Li_x_PF_y_, LiF and Li_2_CO_3_ species. While this type of inorganic CEI is still sufficiently conductive for lithium ion, it has the potential to impede the transport of bulky anionic covalents in the DIB.

**Figure 2:**
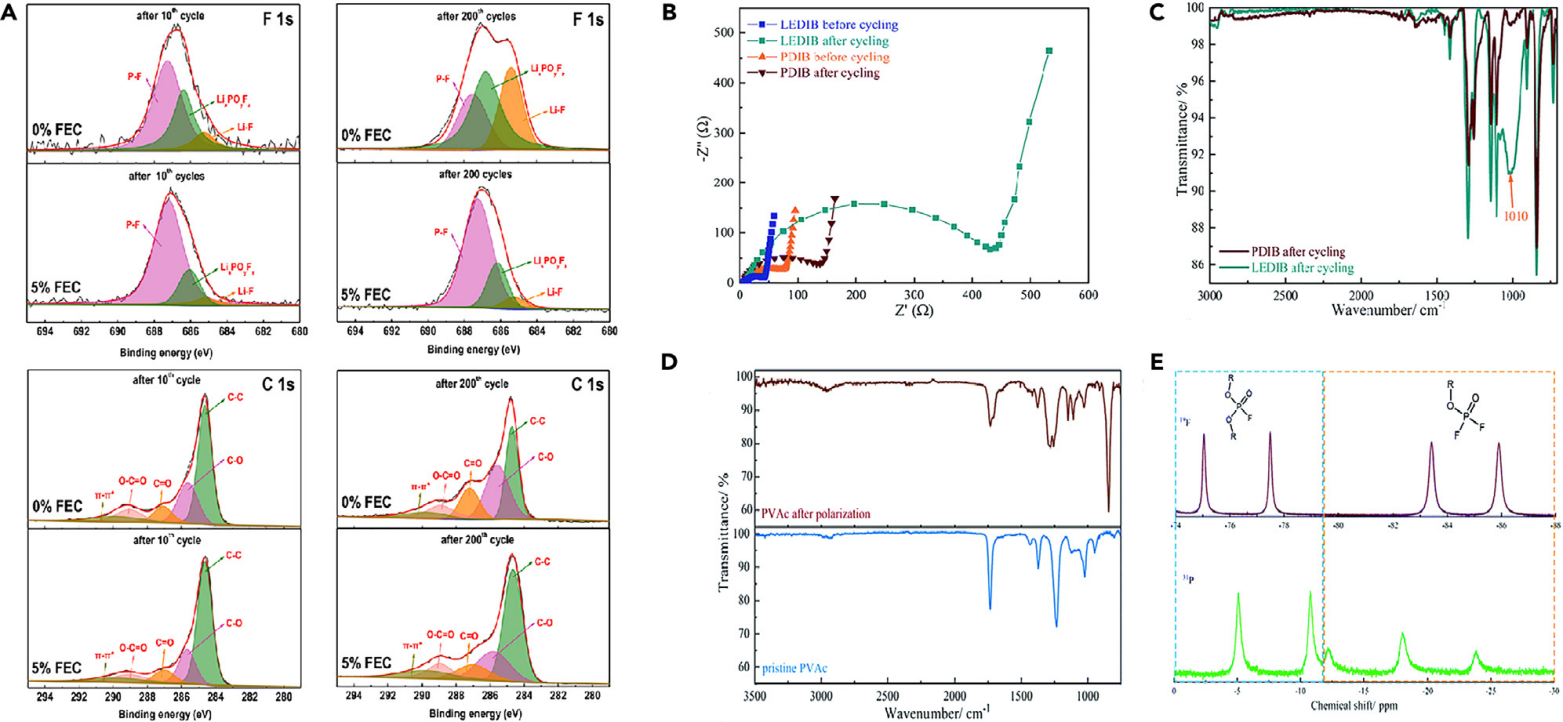
Some spectra from references (A) XPS spectra (F1s and C1s) of graphite cathodes at 5.1 V tested in 0% and 5% FEC-containing electrolytes. Reprinted with permission from Wang et al.^37^ Copyright 2020 Elsevier B.V. (B) The Nyquist plots of the two cathodes before and after cycling. (C) The FTIR spectra of the cycled cathodes. (D) FTIR spectra of pristine PVAc and its derivatives. (E) The ^19^F NMR and ^31^P NMR spectra of the as-obtained derivatives. Reprinted with permission from Han et al.^38^ Copyright 2020 The Royal Society of Chemistry.

In order to understand the effect of FEC additives on the surface chemistry, Wang et al.^37^ investigated the changes in the chemical composition of the CEI layer on graphite cathode with cycling (after the 10th and 200th cycles) by XPS characterization (Figure 2A). The relative fractions of LiF and Li_x_PO_y_F_z_ species were significantly lower with the FEC additive compared to without FEC. This is attributed to less LiPF_6_ salt decomposition on the graphite surface with the FEC additive (reaction 1 and 3).

(Equation 3) $${\text{PF}}_{5}+2x{\text{Li}}^{+}+2xe^{-1}\to {\text{Li}}_{x}{\text{PF}}_{5-x}+x\text{LiF}$$

The decrease in the amount of electronically insulating LiF may result in a lower resistance of the CEI layer, which in turn lowers the electrode resistance and gives the graphite cathode excellent rate capability in the 5% FEC electrolyte. In addition, the CEI formed by 5% FEC was very robust. No significant increase in LiF and Li_x_PO_y_F_z_ content was observed in the graphite cathode using the FEC electrolyte even after 200 cycles compared to the electrodes tested in the 0% FEC electrolyte.

Han et al.^38^ conducted a series of physical characterizations to understand the reasons behind the enhanced cycling performance of the polymer electrolyte in DIB (PDIB). On the cycled graphite positive electrode of PDIB, few by-products from electrolyte decomposition were observed, resulting in particle morphology similar to that of the original graphite. However, the liquid electrolyte DIB (LEDIB) was covered with accumulated by-products, leading to reduced PF_6_^−^ permeation and high impedance (Figure 2B). Fourier transform infrared spectroscopy (FTIR) and XPS spectra of the cycled positive electrode provided insights into the surface chemical compositions of both electrodes (Figure 2C). Considering the decomposition of LiPF_6_, the decomposition mechanism of the electrolyte suggests that despite their similar functional groups, PDIB exhibits weaker C-O characteristic peaks compared to LEDIB, implying significantly reduced EMC oxidation activity. This advantage suppresses excessive growth of the CEI layer, thereby reducing resistance to anion permeation.^39^ Additionally, due to the strong affinity between sulfolane and PF_6_^−^, anion migration characteristics are inhibited, and the viscosity of the electrolyte is not weakened. The formation of CEI mainly comes from sulfolane (SL) and EMC in the solvent.

(Equation 4) $$\text{SL}+{\text{Li}}^{+}\overset{-e^{-}}{\to }{\text{RSO}}_{3}\text{Li}+{\text{RSO}}_{2}\text{Li}$$

(Equation 5) $$\text{EMC}+{\text{Li}}^{+}\overset{-e^{-}}{\to }\text{ROLi}+{\text{ROCO}}_{2}\text{Li}+{\text{Li}}_{2}{\text{CO}}_{3}+{\text{CO}}_{2}↑+H_{2}↑$$

Furthermore, a detailed study of the mechanism of interface self-reinforcement of polyvinyl acetate (PVAc) after introduction was conducted. Based on the above results, it can be speculated that PVAc undergoes some chemical or electrochemical conversion reactions under high voltage, and FTIR and nuclear magnetic resonance spectroscopy were performed on PVAc derivatives obtained from the positive electrode side of the liquid electrolyte solution. Compared to the observation results of the original PVAc, a new characteristic peak appeared at 842 cm^−1^, corresponding to the appearance of the P-F group, revealing the presence of PF_5_ derivatives or residual PF_6_^−^ anions (Figure 2D). Some ester groups in PVAc react with PF_5_ derivatives to form fluorodialkylphosphates and difluoroalkylphosphates. It can be inferred that the formation of CEI also involves the salt in the electrolyte (reaction 6). The ^19^F NMR (376 MHz) and ^31^P NMR (162 MHz) spectra (Figure 2E) confirm the existence of such substances.

(Equation 6) $${\text{LiPF}}_{6}+H_{2}O+{\text{Li}}^{+}\overset{+e^{-}}{\to }\text{LiF}+{\text{Li}}_{x}{\text{POF}}_{y}+\text{HF}$$

Based on these analyses, the possible mechanism of polyvinyl acetate (PVAc) can be speculated as follows. In the liquid electrolyte containing LiPF_6_ and EMC, LiPF_6_ undergoes chemical decomposition, releasing LiF and PF_5_. The latter can react with trace water or ROH species in the electrolyte to generate catalytic POF_3_ intermediates, which then undergo a series of self-catalytic decompositions of LiPF_6_ and EMC. Additionally, under high voltage conditions, H transfer reactions between EMC and PF_6_^−^ or a series of complex EMC cleavage reactions can lead to the continuous formation of ROH species, which then hydrolyze LiPF_6_, further releasing POF_3_ and exacerbating EMC decomposition. Due to these parasitic reactions catalyzed by chemical and electrochemical processes, a large amount of by-products accumulate on the positive electrode surface, eventually leading to rapid failure of the LEDIB. Regarding polymer electrolyte, PVAc exhibits stronger reactivity toward POF_3_ intermediates, thus limiting the kinetics of self-catalytic electrolyte decomposition. Additionally, PVAc derivatives with lowered highest occupied molecular orbital (HOMO) levels can facilitate the interface self-reinforcement process of polymer electrolyte, thereby improving the compatibility of the positive electrode/electrolyte interface. CEI plays an important role in DIBs by stabilizing the graphite positive electrode and improving battery performance and cycling stability. The resources of CEI components are closely related to additives, main salts, solvents, and other factors. By adjusting these factors, the formation and properties of CEI layer can be effectively controlled to optimize the performance of DIBs batteries.

In general, during the process of anion intercalation into the graphene layer, solvent molecules in an electrolyte are solvated around the anions and intercalated into the graphite layer, eventually causing the graphite to exfoliate. The electrolyte also undergoes oxidation and decomposition during the charging process, forming a CEI layer on the graphite cathode. However, due to the repeated intercalation and de-intercalation of anions, the CEI layer continues to fracture and reform as the volume changes, leading to electrolyte consumption and impacting the cycling stability of the graphite. The CEI layer can provide a protective barrier, preventing direct reactions between the electrolyte species and the electrode, thereby reducing electrolyte decomposition and consumption. This helps improve the cycling stability and lifespan of the battery. The ideal CEI layer should be able to regulate the anion conductivity of the battery, facilitate anion migration between the electrolyte and the electrode, and minimize internal resistance. Therefore, the CEI layer should first have good mechanical strength to alleviate stress caused by volume expansion. Additionally, it should possess electrochemical stability and promote the desolvation of anions.

## Strategies for stabilizing the cathode electrolyte interphase layer

To enhance the stability of the CEI layer in GDIBs and prolong its lifespan, while minimizing adverse reactions detrimental to battery performance such as the dissolution of the positive electrode material, electrolyte decomposition, phase transition, and cracking caused by ion intercalation into the positive electrode material, and gas evolution, multiple strategies can be employed. Firstly, by introducing specific additives and binders, the affinity between the electrolyte and graphite cathode can be improved, thereby enhancing the stability of the CEI layer. Secondly, optimizing the composition of the electrolyte, and selecting appropriate salt ions and solvents, can help reduce solvent decomposition and facilitate ion migration. Finally, surface modification of the graphite positive electrode, by introducing functional groups or applying protective coatings, can further improve the stability of the CEI layer. For instance, surface modification of graphite by introducing dopant elements or polymer layers can enhance the migration rate of specific ions within the CEI layer. Hence, further in-depth research and experimental validation are required to optimize and select these strategies to ensure their effectiveness and feasibility in practical applications.

### Electrolyte additives

The addition of additives is the most direct and cost-effective method in the current optimization strategy for stabilizing the CEI layer. Additives with HOMO values higher than solvents and other components can preferentially decompose on the positive electrode surface. Sacrificing the unique structure and functionality of additives can stabilize the electrochemical oxidation at the interface, thus constructing a CEI layer with high ion conductivity and good chemical electrochemical stability. Because additives are only present in trace amounts, they cannot completely replace the electrolyte decomposition process. However, additives can be uniformly dispersed in the electrolyte, thereby adjusting the formation process and elemental composition of the CEI. Classification based on composition includes fluorine-containing additives,^40^^,^^41^^,^^42^ phosphorus-containing additives,^43^^,^^44^ silicon-containing additives.^45^ Some additives in the electrolyte are used to promote the formation of an effective CEI layer during cycling. In particular, some monomers containing ethylene or vinylene moieties, when free radical additives are allowed in the electrolyte formulation, can undergo *in-situ* polymerization on the electrode surface via electrochemical or chemical means. The choice and content of these monomers play a crucial role in the formation of the CEI layer.^34^^,^^37^ In this regard, some typical additives include ethylene carbonate (EC),^46^ FEC,^37^^,^^47^ trimethylsilyl phosphate (TMSP),^22^ lithium difluoro(oxalate)borate salt (LiDFOB),^48^ and so forth, aiming to enhance the performance of the CEI layer on graphite electrodes.

**Figure 3:**
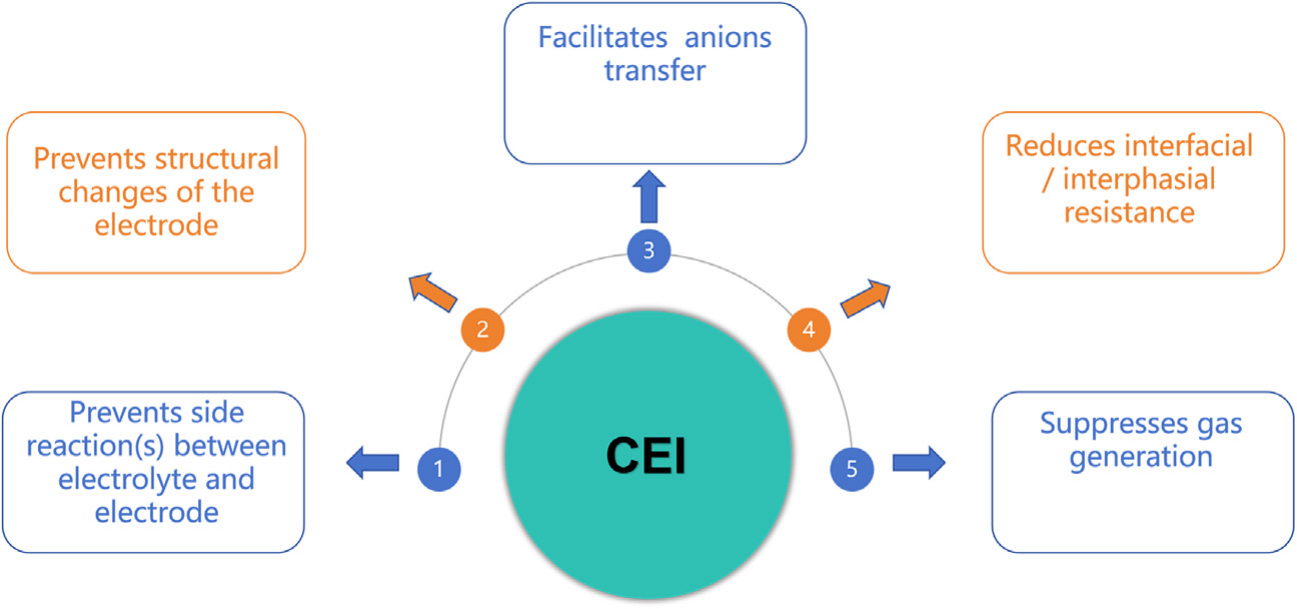
Set of requirements for an ideal CEI Reprinted with permission from P. Kühn et al.^47^ Copyright 2021 Advanced Materials Interfaces published by Wiley-VCH GmbH.

Organic fluorides represent a category of functional additives that have been extensively researched and employed in various applications. Based on the heteroatoms utilized, they can be further categorized into different subgroups. Additionally, two other potential additives are organic metal-based and lithium salt-based molecules, commonly utilized in high-pressure applications. Prior to the current state-of-the-art electrolytes, all these additives undergo decomposition, forming an effective protective film known as the CEI. However, not every formed CEI is an ideal compromise solution. Taking fluorine-containing additives as an example, these additives need to simultaneously meet several requirements (Figure 3). Firstly, they should promote or at least not impede the transport of anions to ensure good battery performance. Secondly, the role of additives should also include reducing interfacial resistance, contributing to enhanced conductivity of the battery. Thirdly, fluorine-containing additives need to decrease the dissolution of transition metals to maintain battery stability. Simultaneously, inhibiting gas generation is another crucial aspect to prevent an increase in internal battery pressure. Furthermore, these additives must prevent structural changes in the active materials to maintain the long-term cycling performance of the battery. Finally, they need to prevent side reactions between the electrolyte formulation and the electrode to ensure good compatibility between battery components.^47^

**Figure 4:**
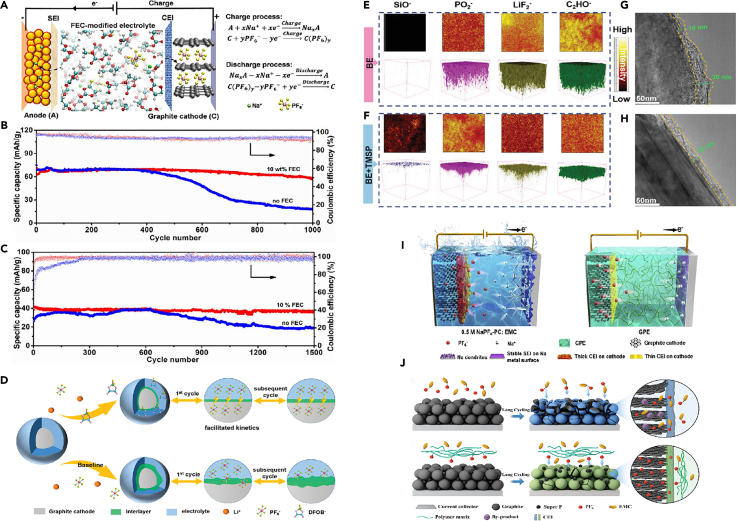
Characterization of SEI and schematic illustrations (A) Working principle and reaction mechanism of graphite cathode-based full batteries. (B and C) Cycling performance of the full batteries of (B) Sn (−)//graphite (+) full cells at 200 mA g^−1^, and (C) NCF (−)//graphite (+) batteries at 500 mA g^−1^, in the voltage window of 2.0–4.8 V when utilizing two Na^+^-based electrolytes, with and without 10 wt % FEC, respectively. Reprinted with permission from Yu et al.^27^ Copyright 2021, American Chemical Society. (D) Illustration of the CEI evolution during cycling in electrolyte with and without LiDFOB additive. Reprinted with permission from Wang et al.^48^ Copyright 2021 Wiley-VCH GmbH TOF-SIMS. (E and F) Investigations of (E) BE, and (F) TMSP in BE induced CEI structures on graphite cathode after 100 cycles. (G and H) HRTEM images of the graphite cathodes after 100 cycles will be and TMSP in BE. Reprinted with permission from Cheng et al.^22^ Copyright 2022 Wiley-VCH GmbH. (I) Schematic illustration of the DISBs using 0.5 M NaPF_6_-PC in EMC liquid electrolyte (left) or GPE (right). Reprinted with permission from Xu et al.^49^ Copytight 2020 Elsevier Inc. (J) Schematic illustration of the EMC co-intercalation behavior comparison in LE and PCME based DIBs. Reprinted with permission from Jiang et al.^50^ Copyright 2022 Wiley-VCH GmbH.

Recent research has demonstrated the effectiveness of fluoride-containing solvents in the formation of stable CEI layers in GDIBs. Wang et al.^37^ investigated the introduction of FEC additive for PF_6_^−^ intercalation in DGIBs. The results show that FEC induces a protective CEI on the graphite cathode, effectively suppressing electrolyte decomposition and stabilizing the graphite surface. This CEI enables outstanding cycling stability with a high coulombic efficiency of 99.0% over 5000 cycles, facilitating ultra-fast charge transfer at the electrolyte-electrode interface and significantly reducing battery resistance. The capacity retention at a cutoff voltage of 5.1 V is impressive at 85.1%, and the CEI layer also minimizes self-discharge. Furthermore, Yu et al.^27^ proposed an innovative fluorinated co-solvent-modified electrolyte tailored for graphite cathodes in sodium-based DIBs. The introduced components participate in the solvation of Na^+^ cations and PF_6_^−^ anions, forming an effective interface layer rich in NaF on the negative electrode. This modification results in two main effects: the formation of a NaF-enriched SEI on the negative electrode effectively suppresses side reactions, and a robust fluorinated solid CEI layer is formed on the graphite cathode, mitigating the oxidative degradation of the electrolyte at high voltages and significantly inhibiting volume changes in graphite during extended cycling (Figure 4A). Compared to electrolytes without FEC (Figures 4B–4C), the FEC-modified electrolyte not only enhances the capacity of the graphite cathode with high charge-discharge efficiency but also maintains an excellent cycle life over 4000 cycles. Additionally, some people have explored the impact of additives such as LiDFOB on the CEI layer on the graphite surface. Wang et al.^48^ successfully formed a thin and robust CEI layer on the graphite cathode by introducing LiDFOB, promoting anion storage reactions, suppressing side reactions, and stabilizing the graphite structure (Figure 4D). They explored the fundamental reasons for the improved performance with the addition of LiDFOB by studying the morphological evolution of the positive electrode in a fully charged state using TEM and SEM. It presents SEM and TEM images of graphite electrodes in electrolytes without LiDFOB and with 0.5 wt % LiDFOB after the 2nd and 150th cycles. For the battery without LiDFOB, a thick and uneven CEI was observed on the material surface after the 2nd cycle, which further increased to ∼56 nm after 150 cycles. This result corresponded to the poorer cycling performance of the electrode material, as a low CE indicates continuous side reactions between the electrolyte and graphite surface, leading to increased CEI thickness and larger interface resistance, hindering the transport of PF_6_^−^ anions to the graphite cathode. In contrast, graphite electrodes cycled in electrolytes containing 0.5 wt % LiDFOB exhibited thin CEI with a thickness of ∼3 nm in both charged and discharged states after the 2nd cycle. After 150 cycles, the CEI remained around 7 nm, indicating enhanced interface stability of the CEI modified by the LiDFOB additive. Additionally, no CEI was observed on the graphite electrode in the electrolyte containing LiDFOB before charging/discharging, suggesting that the CEI is formed by electrochemical reactions between the graphite cathode and the electrolyte. After cycling in the LiDFOB-containing electrolyte, the overall morphology of the graphite remained unchanged, similar to the appearance of the original material. Therefore, the stable CEI formed *in situ* by using the LiDFOB additive can significantly reduce parasitic reactions and improve cycling stability. To elucidate the effect of LiDFOB-induced CEI on the rate capability of graphite cathodes, charge-discharge cycles of DIBs were conducted at current rates ranging from 1 to 50 C, indicating surface structural damage to the graphite material due to high current rates, leading to the exposure of fresh graphite surfaces to electrolytes with higher reactivity and lower CE, consistent with SEM and TEM analyses. These results suggest that the LiDFOB additive plays a dominant role in generating a protective and low-resistance CEI, contributing to highly improved interface kinetics. In comparison of the activation energies in electrolytes with and without LiDFOB, the activation energies of graphite batteries were 42.1 and 57.9 kJ mol^−1^, respectively. The use of electrolytes with LiDFOB resulted in lower activation energy, indicating that PF_6_^−^ ions migrate more easily through the LiDFOB-induced CEI, significantly enhancing the rate capability of the battery and reducing electrode polarization compared to batteries without the additive. The thin and robust CEI formed by adding LiDFOB not only suppresses adverse side reactions between the electrolyte and the positive electrode but also stabilizes the graphite cathode, preventing structural degradation or peeling, thereby contributing to excellent cycling performance. This allows the graphite||lithium battery to maintain 87.5% capacity after 4000 cycles at 5 C and 88.8% capacity after cycling at 40 C (4 A g^−1^), providing high power of 0.4–18.8 kW kg^−1^ at an energy density of 422.7–318.8 Wh kg^−1^. This innovative approach significantly improves the performance of DIGBs by suppressing adverse reactions at the electrolyte-graphite interface, enhancing cycling performance, maintaining high energy efficiency, and prolonging battery lifespan. It is noteworthy that there is currently no consensus on whether FEC contributes to the formation of the positive electrode CEI layer. Some studies suggest that fluorinated monomers may stabilize the CEI layer, while others propose that these monomers, when used with salt additives, may lead to the formation of unstable fluorine substances during cycling processes. Further investigation is needed to clarify this aspect.^46^

Cheng et al.^22^ reported a electrolyte additive for DIBs, namely TMSP, and extensively studied its role in enhancing electrochemical stability and kinetic performance. The results showed that, as shown in (Figures 4E and 4F), it is easy to see that the overall thickness of the CEI on graphite cycled with TMSP is much smaller than that on graphite cycled without TMSP. It was found that a thick and rough CEI layer covered the graphite cathode cycled in the base electrolyte (Figure 4G). This CEI layer can reach 30 nm. The graphite cycled in the electrolyte containing TMSP was uniformly covered by a thin CEI layer (∼12 nm) (Figure 4H). After adding TMSP, the CEI of the battery became thinner, and the impedance of the battery also decreased. The study revealed that during the cathode process, TMSP decomposes prior to EMC and PF_6_^−^, thereby acting as an oxidant to improve the physical and chemical properties of the CEI layer on the graphite cathode. TMSP, with appropriate chemical composition (enriched with SiO^−^ and PO_2_^−^), significantly enhances the structural stability of graphite and suppresses various side reactions. Considering the effectiveness, convenience, and low cost of TMSP as a multifunctional electrolyte additive, this study provides important insights into improving the performance of DIBs with *in-situ* CEI structure on graphite cathode.

Xu et al.^49^ reported a high-performance DIB utilizing a multifunctional gel polymer electrolyte (GPE). This GPE was synthesized *in situ* by thermopolymerizing ethoxylated pentaerythritol tetraacrylate (EPTA) monomers in an optimized electrolyte containing FEC as a cosolvent and 1,3-propane sultone (PS) as an additive (Figure 4I**)**. To further analyze the surface composition of CEI, XPS depth profiling measurements of unoptimized electrolytes and GPEs were compared. XPS results showed that in 0.5 M NaPF_6_-PC:EMC liquid electrolyte, the CEI on the graphite cathode mainly consists of abundant alkyl sodium carbonate (ROCO_2_Na) and polycarbonate, Na_x_PO_y_F_z_ and NaF produced by NaPF_6_ decomposition, and a large amount of PF_6_^−^. Importantly, the intensity of PF_6_^−^ rapidly decreases with increasing sputter depth and almost disappears at depths exceeding 20 nm. This verified that in 0.5 M NaPF_6_-PC:EMC electrolyte, non-uniform PF_6_^−^ anion flux cannot penetrate thick CEI and intercalate into the graphite cathode, consistent with *in situ* XRD results. In contrast, for GPE-based batteries on fully charged graphite cathode surfaces, a new F 1s peak at around 686.5 eV is attributed to FEC decomposition products (-CHF-OCO_2_-) drifting from the negative electrode, while the O1s peak at around 532.5 eV is attributed to oxidized products of PS addition, C-S-O. Fluorine-containing species (-CHF-OCO_2_-) and alkyl sulfonate (C-S-O) in the CEI effectively suppress electrolyte decomposition, reducing CEI thickness. Moreover, even at a sputter depth of 160 nm, the F1s and P2p spectral peaks. Jiang et al.^50^ also designed a polymer electrolyte with 2, 2-azobisisobutyronitrile (AIBN) additive to preserve the oxidative stability of the cathode, and anionic desolvation was important for the effective inhibition of solvent co-intercalation layer and oxidative decomposition of the electrolyte, which resulted in the improvement of CEI (Figure 4J). They designed a polymer electrolyte, termed “anion-permselective” polymer electrolyte (thereafter denoted as PCME), containing a cationic quaternary ammonium functional group (-N(CH_3_)^3+^), where the PF_6_^−^ anion is decoupled from the EMC solvent to facilitate desolvation and subsequent intercalation into graphite. Here, the selective intercalation of PF_6_^−^ by the polymer electrolyte in the absence of EMC resembles the selectivity of the cell layer, which only allow certain molecules to pass. Through strong electrostatic interactions, the PF_6_^−^ anion in PCME preferentially interacts with the dangling cationic chains N(CH_3_)^3+^ rather than coordinating with EMC, thereby weakening the interaction between PF_6_^−^ and EMC and promoting PF_6_^−^ desolvation. It was found that modulating the solvation structure of PF_6_^−^ can alleviate EMC co-intercalation and maintain the integrity of the graphite structure. Simultaneously, the decreased affinity concentration of PF_6_^−^-EMC also enhanced the oxidative stability of the EMC-based electrolyte. Consequently, the assembled battery significantly extended cycling performance, with a capacity retention of 87.1% after 2000 cycles and an average CE of 99.0%. Furthermore, another benefit of the polymer electrolyte design is that the polymer matrix can construct a stable CEI, contributing to improved cycling performance and CE. DIBs based on PCME exhibit a larger initial irreversible capacity, indicating the evolution of the polymer matrix on the graphite surface. XPS analysis of the graphite cathode surface after 300 cycles reveals a reduced peak intensity of P-O and CO_3_^2−^ species in the CEI when PCME is used, demonstrating less electrolyte decomposition compared to the baseline electrolyte. Additionally, higher content of C=O species and abundant nitrogen species (C-N, N-P, and so forth) are observed on the cathode surface with PCME compared to the baseline electrolyte, indicating the involvement of the polymer matrix in constructing an interface organic-inorganic hybrid layer. This suggests that the aminoethyl ester groups on PCME can capture alkylphosphate derivatives generated during LiPF_6_ decomposition in high-voltage cycling, resulting in mechanically stable CEI, preventing continuous electrolyte component decomposition. High-resolution transmission electron microscopy (HRTEM) and Young’s modulus analysis confirm the mechanically stable CEI induced by PCME. The lower Young’s modulus of the cyclic cathode surface indicates that the CEI has high elasticity to withstand volume stress during cycling. In conclusion, the polymer-enhanced CEI improves the long-term cycling performance of DIBs to a certain extent. It should be noted that selecting appropriate electrolyte additives requires the consideration of the performance requirements and application scenarios of the battery, and reasonable proportioning and optimization should be conducted. Additionally, the selection and proportioning of additives also need to be fully studied and evaluated to ensure their positive impact on battery performance.

**Figure 5:**
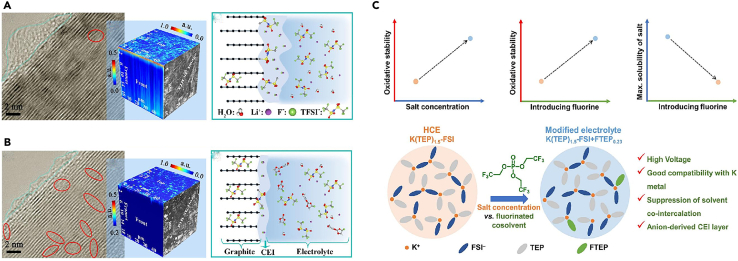
Structural characterization for graphite cathode (A and B) High resolution transmission electron microscopy (HRTEM) images of fully charged graphite in pristine and FEC-added electrolytes, respectively. Time of flight secondary ion mass spectrometry (TOF-SIMS) pattern of Li element on graphite in pristine and FEC-added electrolytes. The plane of Top and Front are graphite surface and perpendicular to graphite surface (The scale of depth is frames), respectively. Schematic illustration of TFSI– ions intercalated into graphite cathode in pristine and FEC-added electrolytes. Reprinted with permission from Zheng et al.^51^ Copyright 2024 Elsevier B.V. All rights reserved. (C) Schematic illustration showing the impacts of salt concentration and fluorinated cosolvent on the electrolyte oxidative stability. The solvation structure of HCE and the electrolyte design principle for graphite cathode-based DIBs. Reprinted with permission from Luo et al.^52^ Copyright 2024 Elsevier Ltd. All rights reserved.

Zheng et al.^51^ demonstrated the impact of FEC additive on the formation of the CEI through HRTEM. In the original electrolyte, the CEI appeared thicker due to the presence of amorphous regions at the graphite edges (Figure 5A). However, in the electrolyte with added FEC, the CEI became thinner and more uniform (Figure 5B**)**. This aligns with the results from Raman spectra, indicating that optimized FEC electrolyte can suppress the formation of the CEI during charging. Additionally, compared to the original electrolyte, the (002) lattice planes of graphite in the FEC-added electrolyte exhibited more defects. This suggests that a relatively uniform and light CEI facilitates more TFSI^−^ ions intercalating into the graphite layers, leading to more defects on the (002) lattice planes. TOF-SIMS testing tracked the distribution of lithium ions, consistent with XPS results. In the direction perpendicular to the graphite surface, lithium ions in the original electrolyte were mainly found within the depth range of 0–1.2 frames, whereas trace amounts of lithium ions were observed in the FEC-added electrolyte. This further illustrates that the addition of FEC can suppress the formation of surface CEI. During charging, TFSI^−^ ions migrate from the electrolyte to the graphite electrode, resulting in the presence of lithium ions on the graphite surface due to dissolution. Dissolved TFSI^−^ ions decompose to produce LiF, thereby forming a thick and irregular CEI on the graphite surface, preventing the rapid intercalation of TFSI^−^ ions into the graphite cathode at higher potentials. Consequently, the entire graphite electrode exhibits higher activation energy and polarization effects, resulting in a discharge capacity of only 22 mAh g^−1^. In the FEC-added electrolyte, the activity of F atoms in TFSI^−^ was reduced due to the addition of FEC. Furthermore, FEC was often enveloped outside water and lithium ions, forming a solvate structure. FEC plays a protective role in the solvate structure of TFSI^−^ and inhibits the decomposition of TFSI^−^ due to its antioxidative properties, thereby forming a thin and uniform CEI at higher potentials. This facilitated the rapid intercalation of TFSI^−^ ions from the electrolyte into the graphite cathode, resulting in a higher specific discharge capacity (47 mAh g^−1^) and lower polarization effects. Additionally, TOF-SIMS was used to track the presence of F elements on the graphite surface and perpendicular to the graphite surface. In areas with graphite edges or significant defects, the accumulation of F elements is more prominent, while only trace amounts of F elements can be detected in relatively flat regions. This result suggested that CEI is more likely to form at graphite defects or edges. A significant F signal was detected within the range of 1–1.2 frames on the front face in the original electrolyte, consistent with the detection of Li elements, both of which belong to the CEI signal. As the depth of ion beam cutting increases, there is no prominent F element signal. However, with an increase in the depth of ion beam cutting, the F element signal can be detected in the FEC electrolyte. This once again demonstrated that reducing the thickness of the surface CEI is conducive to the intercalation of TFSI^−^ ions into graphite to form graphite intercalation compounds. Luo et al.^52^ suggested that, generally, the oxidative stability of electrolytes increases with salt concentration (Figure 5C**)**. This was attributed to a transition in the solvation structure from solvent-separated ion pairs to coordinated ion pairs and ion-aggregate structures. The addition of fluorinated solvents typically confers higher oxidative potentials compared to non-fluorinated solvents because the introduction of electron-withdrawing fluorine atoms can lower the HOMO energy level of solvent molecules, thus imparting greater electrochemical stability to the electrolyte at the cathode side. Additionally, they can decompose into compounds containing fluorine to enhance the chemical and mechanical stability of the electrode/electrolyte interface.

Overall, many researches supported the conclusion that some kind of additives positively impact the CEI layer. The additives to improve the performance of CEI are summarized in Table 1. The use of additives containing fluorine, silicon, phosphorus, and other elements may have various effects on the CEI layer of DGIBs.^22^^,^^27^^,^^34^^,^^48^^,^^53^^,^^54^ Introducing fluorine compounds and silicon compounds as additives enhance the stability and durability of the CEI layer, forming a more robust interface, reducing electrolyte decomposition, and corrosion of graphite, thereby improving the cycle life and long-term performance. Furthermore, the phosphorus compounds have been studied to suppress the possibility of adverse reactions in the CEI, as well as fluorine, silicon, phosphorus, and other additives were utilized to optimize the interaction between the electrolyte and graphite, promoting more efficient charge transfer and increasing the battery’s energy density. The introduction of these additive elements also alters the chemical composition of the CEI layer, leading to the emergence of new chemical reaction pathways, thereby affecting interface stability and electrolyte degradation mechanisms.

**Table 1:** Several additives for improving the CEI and electrochemical performance

Additive	Cathode	Electrolyte	Voltage range (V)	Capacity (mAh g^−1^)	Year and ref.
FEC	graphite	3 M LiPF_6_ in FEC:EMC (1:20 by volume)	5.1–3.0	77 (500mA g^−1^)	Wang et al., 2020^37^
FEC	composite graphite	1 M LiPF_6_ in EC:DEC (1:1 by volume)+5vol % FEC	5.0–2.0	65 (100mA g^-1^)	Ghosh et al.,2023^32^
FEC	graphite	3 M NaPF_6_ in EMC:PC, (1 : 1 by volume)+ 10 vol % FEC	5.0–3.0	92.7 (2.0 A g^−1^)	Guo et al., 2022^55^
FEC	graphite	1 M LiPF_6_ in FEC:FEMC (3：7 by volume)	5.2–3	88.7 (500 mA g^−1^)	Wang et al., 2022^56^
FEC	graphite	1 M NaPF_6_ in EC:DMC:EMC (1:1:1 by volume)+10wt % FEC	4.8–2	38.7 (500 mA g^−1^)	Yu et al., 2021^27^
FEC	graphite	1 M NaPF_6_ in PC +5wt % FEC	4.5-1.5	58.5 (100 mA g^−1^)	Yang et al., 2023^57^
FEC	composite graphite	1 M LiPF_6_ in EC:DEC:EMC (1:1:1 by volume)+2 vol % FEC	4.5-2.0	74.9 (2000 mA g^−1^)	Li et al., 2020^58^
FEC	graphite	3 M NaPF_6_ in EC: EMC (1 : 1 by volume)+ 10 wt % FEC	5.0–3.0	65 (1A g^−1^)	Hou et al., 2021^59^
LiDFOB	graphite	3 M LiPF_6_ in EMC +0.5 wt % LiDFOB	5.1–3.0	81 (500 mA g^−1^)	Wang et al., 2021^48^
LiDFOB	graphite	2 M LiPF_6_ in FEMC +1.0 wt % LiDFOB	3.7-2.0	85 (20 C)	Zhu et al., 2024^60^
VC	graphite	3.5 M LiPF_6_ in EMC+2 wt % VC	5.0–3.4	54 (50 mAg^−1^)	Heidrich et al., 2019^61^
VC	graphite	4 M LiPF_6_ in EMC +3 wt%VC	5.0–1.0	104 (0.5C)	Wu et al., 2019^62^
VC	graphite	4.0 M LiPF_6_ in EMC +2.0 wt % VC	5.0–3.0	90 (100 mA g^−1^)	Wang et al., 2019^63^
VC	graphite	4.0 M LiPF_6_ in EMC +5 wt % VC	5.0–3.0	93 (2 C)	Tong et al., 2016^64^
VC	graphite	4 M LiPF_6_ in EMC +5wt % VC	4.95-3.0	130.3 (1 C)	Song et al., 2019^65^
VC	graphite	4.0 M LiPF_6_ in EMC +5 wt % VC	5.0–3.0	88 (15 C)	Tong et al., 2017^66^
VC	graphite	4 M LiPF_6_ in EMC +2 wt % VC	4.8-2.0	125 (1 C)	Wang et al., 2019^67^
VC+ LiNO_3_	graphite	4 M LiPF_6_ in EMC+3vol % VC+0.03 M LiNO_3_	5.0–3.4	69 (5 C)	Wu et al., 2019^68^
VC+ AlF_3_	graphite	3M LiPF_6_ in EMC:DMC (1:1 by weigh)+2wt % AlF_3_ +3wt % VC	5.0–3.0	100 (200 mA g^−1^)	Wang et al., 2017^69^
VC+ FEC+ LiPF_6_	graphite	4 M LiPF_6_+ 0.1 M LiFSI+EMC + 3vol % VC+5 vol % FEC	5.0–3.0	95 (2 C)	Wu et al., 2022^70^
ES	graphite	0.3 M KTFSI in Pyr_14_TFSI +2 wt % ES	5.0–3.0	42 (250 mA g^−1^)	Beltrop et al., 2017^71^
ES	graphite	0.3 M NaTFSI in Pyr_14_TFS +2 wt % ES	4.7-3.0	46 (50 mA g^−1^)	Meister et al., 2017^72^
ES	graphite	1 M LiTFSI in Pyr_14_TFSI +2 wt % ES	5.2–3.0	121 (10 mA g^−1^)	Rothermel et al., 2014^73^
TMSP	composite graphite	3 M LiPF_6_ in EMC+3wt % TMSP	5.0–3.0	71.9 (5 C)	Cheng et al., 2022^22^
PS	graphite	0.5 M NaPF_6_ in PC:EMC:FEC (1:1:1 by volume) +4 wt % PS+1.5 wt % EPTA+0.1 wt % AIBN	3.0–2.0	78 (100 mA g^−1^)	Xu et al., 2020^49^
TAP	graphite	6 M KTFSI in DMC +2 wt % TAP	4.5-1.0	67 (20 mA g^−1^)	Asfaw et al., 2022^74^
DAP+ PETEA	graphite	1 M LiPF_6_ in FEC: FEMC: HTE (1: 6: 3 by volume)+3 wt % DAP +1.5 wt % PETEA	5.0–3.0	83.5 (1 C)	Wu et al., 2021^75^

### Binders

In addition to electrolyte additives, enhancing the stability of the CEI layer in DIBs can also be achieved through the addition of functional binders.^76^^,^^77^ Binders that provide sufficient coverage on the surface of active materials can inhibit solvent decomposition and alleviate solvent co-intercalation, which in graphite can lead to detrimental exfoliation.^78^^,^^79^ However, there are a little research focusing on the binders of graphite cathodes in DIBs. Huesker et al.^80^ indicated that carboxymethyl cellulose sodium (CMC) coatings exhibited greater expansion compared to polyvinylidene fluoride (PVDF)-based coatings, resulting in particle detachment upon extended cycling. Chan et al.^81^ investigated the binder-dependent power characteristics of GDIBs. Graphite cathodes based on CMC outperformed those based on PVDF in rate capability tests (83 mAh g^−1^ vs. 43 mAh g^−1^ at 500 mA g^−1^), leading to the conclusion that fluorinated binders increase interface resistance. Kotronia et al.^82^ comprehensively studied the role of binders in the overall performance of DIBs. Substituting CMC with polyvinylidene fluoride-hexafluoropropylene (PVDF-HFP) improved the interface stability of graphite cathodes in DIBs. Electrochemical tests combined with XPS and operational pressure measurements highlighted that PVDF-HFP suppressed parasitic reactions at the CEI, contrasting sharply with CMC. However, CMC induced lower interface resistance, thus being beneficial in terms of rate capability. The use of fluorinated polymer adhesives had a positive impact on CEI formation. Particularly, PVDF binder participated in establishing CEI, with a significant manifestation of passivation films on electrodes (such as graphite) when used in large quantities. Nevertheless, 10%–14% PVDF binder was sufficient to manufacture electrodes with high energy density and a long life cycle. Moreover, the addition of other types of polymer binders can increase the adhesion between graphite cathode materials and electrolyte, promoting the formation and stability of CEI layers. Common polymer binders include polyphosphoric acid (PPA),^83^ polyacrylic acid (PAA),^84^ and so forth.

**Figure 6:**
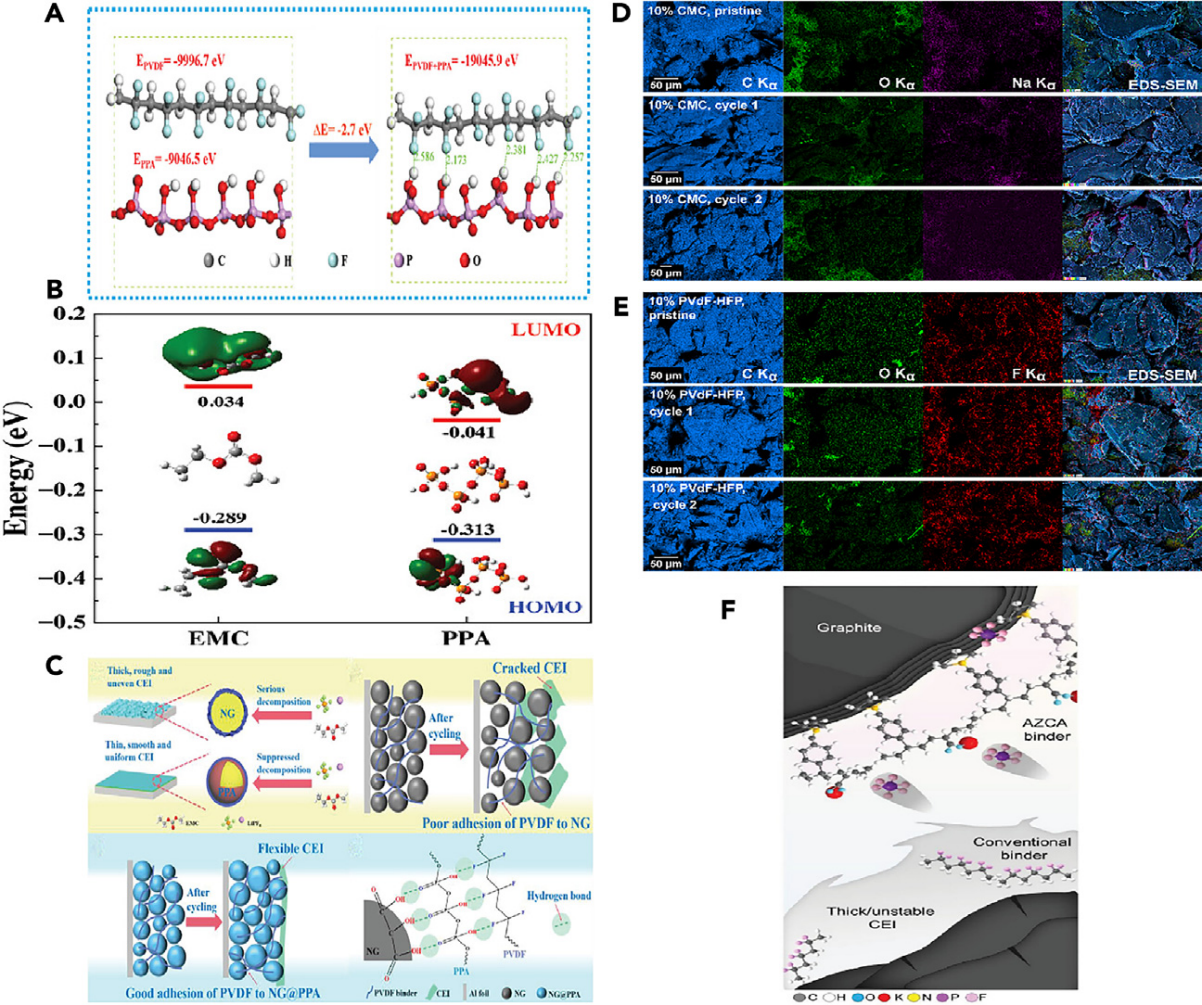
Calculations and schemes (A and B) The DFT calculations on the (A) binding energy between PVDF and PPA, and (B) HOMO and LUMO energies of EMC and PPA. (C) Schematic illustration of the function mechanism of PPA layer on natural graphite (NG) cathodes. Reprinted with permission from Zhang et al.^83^ Copyright 2022 Wiley-VCH GmbH. EDS elemental maps revealing the initial binder distribution and its evolution upon subsequent cycling. (D) Maps of carbon, oxygen, and sodium are provided for the CMC-based electrodes together with the corresponding overlayed EDS-SEM images. (E) maps of carbon, oxygen, and fluorine are shown for PVDF-HFP based electrodes. In both cases, a homogeneous binder distribution was initially observed throughout the entire electrode. Reprinted with permission from Kotronia et al.^82^ Copyright 2023 The Authors. Published by Elsevier B.V. (F) Schematic of graphite electrodes using the designed AZCA binder compared with conventional binder. Reprinted with permission from Kang et al.^85^ Copyright 2023 Advanced Materials published by Wiley-VCH GmbH.

In other study, for example, Zhang et al.^83^ utilized ultrathin PPA film and chemically anchored it uniformly and tightly onto the surface of graphite particles through interfacial hydrogen bonding. This artificially prepared PPA shell contributed to the formation of a fine, smooth, and flexible CEI, effectively reducing electrolyte decomposition. During high-voltage cycling (5 V vs. Li/Li^+^), this shell successfully protected the internal graphite from structural degradation. DFT calculations showed that the fluorine atoms of chain-like PVDF and the hydrogen atoms of PPA could spontaneously form hydrogen bonds (Figure 6A), exhibiting high mechanical rigidity and maintaining electrode structure integrity. The HOMO and lowest unoccupied molecular orbital (LUMO) energies of EMC and PPA (Figure 6B). The HOMO energy roughly evaluates the electron loss capability or its antioxidant ability. PPA had a much low HOMO energy compared to the EMC solvent, implying it would be oxidized at a higher oxidative potential. It can be inferred that PPA on the natural graphite (NG) cathode exhibited electrochemical stability at the high operating potential and successfully mitigated electrolyte oxidative decomposition. It summarized the significantly improved structural stability of the NG@PPA cathode during long-term cycling (Figure 6C), attributed to the PPA coating suppressing electrolyte decomposition, forming a protective CEI layer, and enhancing the mechanical strength of the binder, thereby improving the performance and cycle life of the NG cathode. Interestingly, experimental evidence demonstrated strong interactions between PPA and the binder PVDF, primarily through intermolecular hydrogen bonding. As PPA acted as a bridge between the internal graphite particles and the surrounding PVDF binder, chemical bonding occurred, significantly enhancing the mechanical stability of the graphite cathode. This interaction led to accelerated ion intercalation/de-intercalation kinetics, resulting in a considerably long cycle life of the graphite cathode. Zhao et al.^84^ conducted an in-depth systematic study by mixing graphite and PAA as a positive electrode for DIBs, investigating its thermal stability in the electrolyte. The heat generated by the graphite cathode was influenced by the amount of PF_6_^−^, while the positive electrode-electrolyte interface (stabilized by the binder to form a stable CEI layer) exhibited endothermic properties for all charging states. This differs significantly from traditional LIB cathodes, such as LiCoO_2_, which typically release heat at high temperatures through reactions with the electrolyte. The results also indicated that C_n_(PF_6_) is more stable compared to lithiated graphite LiC_6_. This finding emphasizes the advantages of DIBs in terms of thermal stability, making them a promising candidate for future large-scale applications.

Apart from electrolyte additives, functional binders that enhance the stability of the CEI are also of interest to DIBs. Binders can generate sufficient coverage of active material surfaces to suppress solvent decomposition and alleviate solvent co-intercalation,^78^^,^^79^ which can cause destructive delamination within graphite. However, there has been limited research focused on binders for graphite cathodes in DIBs. Chan et al.^81^ investigated the power characteristics of DIBs with varying binders. In rate capability tests, graphite cathodes based on CMC outperformed those based on PVDF, suggesting that fluorinated binders increase interface resistance. Kotronia et al.^82^ explored the relationship between two commonly used binders, CMC and PVDF-HFP, and CEI stability. XPS analysis revealed a decrease in the relative intensity of C-O, O-C-O, and C(=O)O signals in CMC-based electrodes compared to the graphite matrix. Indeed, after the 125th discharge, the graphite signal was strongest in the C1s spectrum, indicating that the organic component of the CEI is thin and unevenly distributed. The continuous decrease in binder-related signals in the C1s core-level spectrum suggested the partial degradation of CMC, likely due to electrochemical and mechanical processes. Additionally, an additional peak appeared in the F1s spectrum of several samples, indicating the growth of metal fluorides (potentially LiF and AlF_3_) on the graphite cathode and Al current collector, possibly due to the partial decomposition of FSI^−^ anions. Conversely, cyclic electrodes containing PVDF-HFP exhibited an increase in C1s signals associated with binder and decomposed electrolyte, indicating the formation of an organic CEI. Minor variations were observed in the F1s spectrum, suggesting the formation of metal fluorides similar to those in CMC-based electrodes. Energy dispersive X-ray spectroscopy (EDS) imaging was used to track the morphology of graphite cathodes and the distribution of binders (Figures 6D and 6E**)**. Generally, cyclic graphite electrodes with either binder exhibited significant expansion, particularly pronounced at the edges of the flakes. EDS mapping revealed a uniform distribution of CMC within both pristine and cyclic electrodes, while selective evidence of PVDF-HFP was strongest around the contours of graphite particles. Overpotential trends and electrochemical impedance spectroscopy (EIS) indicated that interfaces formed by PVDF-HFP were more resistive than those formed by CMC, thereby suppressing side reactions and self-discharge. Surface analysis and operating pressure measurements suggested that the CEI stability of graphite cathodes using CMC was poorer compared to those using PVDF-HFP.

Kang et al.^85^ proposed an innovative polymer binder design for DIBs and cohesive graphite positive electrodes to achieve excellent stability under high-voltage conditions (>5 V). This innovative binder comprises acrylic ester segments, which, compared to traditional PVDF and polyacrylic ester binders, form deeper and reversible anion intercalation with graphite using a nitrogen-containing compound (represented by AZCA binder), thereby providing higher capacity, durability, and sustainable anion storage (Figure 6F**)**. The high-voltage stable binder forms a stable interface between the graphite positive electrode and the electrolyte, forming a solid CEI layer, ensuring excellent oxidative stability and self-healing properties. The azide portion allows the formation of nitrogen-ring covalent bonds with graphite and interchain cross-linking. Through simple 1-h UV treatment, the binder can be fixed within the electrode, forming covalent bonds with graphite and establishing a robust three-dimensional network. This modification aids in achieving deeper and reversible anion intercalation, thereby enhancing capacity, extending lifespan, and achieving sustainable anion storage.

However, there are also many issues with adhesives in practical applications. For example, traditional electrode composite materials composed of active material powders, polymer binders, and additives are prone to delamination from rigid current collectors during continuous deformation processes, adversely affecting the flexibility of the device. This dilemma has become a major obstacle to the widespread adoption of batteries. Additionally, due to the high aspect ratio of one-dimensional fiber structures, the electron transport in one-dimensional fiber electrodes is significantly focused. Possible material aggregation easily occurs on the curled fiber surface, thereby minimizing surface energy and leading to a decrease in electrochemical performance. Huesker et al.^80^ researched suitable electrode binders to address the significant electrode volume changes of graphite anodes during high-performance DIBs anion intercalation and deintercalation processes, aiming to enhance the mechanical stability of graphite cathode. Long-term expansion and contraction measurements conducted via *in situ* electrochemical dilatometry (ECD) revealed distinct behaviors between electrodes containing PVDF and those based on CMC. While the maximum electrode thickness remained constant for PVDF-containing electrodes, CMC-based electrodes exhibited an initial increase in thickness followed by a decrease after reaching the maximum thickness value. This difference may stem from the varying bonding mechanisms and flexibility of CMC and PVDF. PVDF, owing to its higher flexibility and interaction with graphite surfaces via hydrogen bonding or van der Waals forces, appeared capable of effectively interconnecting all electrode components and preventing particle detachment. In contrast, the lower flexibility of CMC (with a Young’s modulus approximately 6–7 times higher than PVDF) and its bonding to graphite surfaces via hydrogen or covalent bonds could lead to crack formation and active material particle detachment within ECD battery devices, exacerbating capacity decay. Furthermore, electrodes containing CMC exhibited greater expansion compared to those containing PVDF; at 20°C, the electrode thickness change for CMC-containing electrodes reached 131%, whereas PVDF-containing electrodes only increased by 92%. Combining styrene-butadiene rubber (SBR) with CMC could mitigate electrode thickness decay after reaching the maximum thickness and prevent further thickness reduction after 20 cycles, although SBR did not further improve capacity decay. Increasing CMC content to 10 wt % could also attenuate electrode thickness decay but could not completely prevent it. However, higher CMC content could suppress capacity decay within the first 50 cycles. In practical applications, the high cost of polymer electrolytes should be carefully considered concerning their market applicability.

Other types of binders, such as nanoparticle binders such as silica and alumina nanoparticles,^86^^,^^87^^,^^88^ are widely employed in battery technology. They effectively increase the contact area and adhesion between the graphite positive electrode material and the electrolyte by filling and increasing the surface roughness of the positive electrode material, thereby improving battery performance. On the other hand, carbon-based binders, such as carbon black and graphene,^89^^,^^81^^,^^90^^,^^91^^,^^92^ not only enhance the conductivity and electrochemical stability of the graphite positive electrode material but also promote the formation and stability of the CEI layer. These carbon-based binders play a role in connecting and enhancing material performance in batteries, improving battery cycle life and electrochemical performance. Different binders can regulate the interactions between the graphite positive electrode material and the electrolyte, thereby improving the formation and performance of the CEI layer, and enhancing the energy density and cycle life of the battery. Therefore, it should be noted that the selection of suitable binders requires the consideration of their compatibility with the CEI layer, electrolyte, and other battery components, and thorough experimentation and evaluation are needed to determine the optimal binder selection, usage methods, and ratios.

### Optimizing the electrolyte: Main salts and main solvents

In DIBs, binders play a crucial role in electrode fabrication as they not only bind the active material particles and conductive additives but also affect the mechanical stability and ion transport properties of the battery. Simultaneously, the selection of electrolyte, particularly the optimization of the primary salt and solvent, directly influences the ionic conductivity of the electrolyte and its interaction with electrode materials. There exists a close correlation between the selection of binders and the optimization of electrolyte in improving the CEI. Therefore, when designing DIBs, it is essential to consider not only the performance of binders but also the characteristics of the electrolyte. By optimizing the selection of electrolytes, better battery performance can be achieved, including enhanced cyclic stability, higher charge/discharge capacity, and faster charge/discharge rates. Optimizing the electrolyte is a crucial step in DGIBs, directly impacting the performance, stability, and cycle life of the battery. In the electrolytes, the main components include the main salts and main solvents, which collectively act on the CEI layers.^92^^,^^93^^,^^94^^,^^95^^,^^96^^,^^97^

#### Main salts

The electrolyte serves as the conductive substance in the battery, responsible for the transmission of ions between the positive and negative electrodes. Salts within the electrolyte act as carriers for ions, assisting the movement of cations and anions between the two poles of the battery. The ion conduction forms the basis of the electric current in the battery, enabling the battery to undergo the charging and discharging processes. Different types of ions acting on the positive electrode during charging and discharging lead to the formation of different CEI layer materials, affecting the migration rate of ions. Some common main salts include alkali metal salts: commonly used alkali metal salts include lithium salts (such as LiPF_6_,^98^^,^^99^ LiClO_4_; ^46^^,^^100^ LiBF_4_^101^), sodium salts (such as NaPF_6_,^93^^,^^102^ NaClO_4_^103^), potassium salts,^94^^,^^104^ and others.^53^ These salts can provide the ion concentration required for ion transport and exhibit good solubility and electrochemical stability.

**Figure 7:**
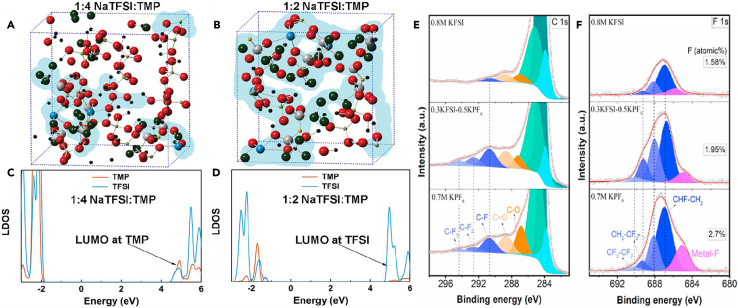
Calculations and XPS spectra (A–D) Density functional theory-molecular dynamics (DFT-MD) simulation of (A) the 1:4 NaTFSI:TMP and (B) 1:2 NaTFSI:TMP electrolytes, atom color: Na, blue; O, red; S, gray; N, yellow; F, cyan; P, light green; C, black; and (C), (D) corresponding local density of states (LDOS). Reprinted with permission from Jiang et al.^105^ Copyright 2018 WILEY-VCH Verlag GmbH & Co. KGaA, Weinheim. (E) C 1s and (F) F 1s XPS surface profiles of graphite electrodes cycled with 0.8 M KFSI, 0.3 KFSI-0.5 KPF_6_, and 0.7 M KPF_6_. Reprinted with permission from Tan et al.^107^ Copyright 2021 Elsevier Ltd.

**Figure 8:**
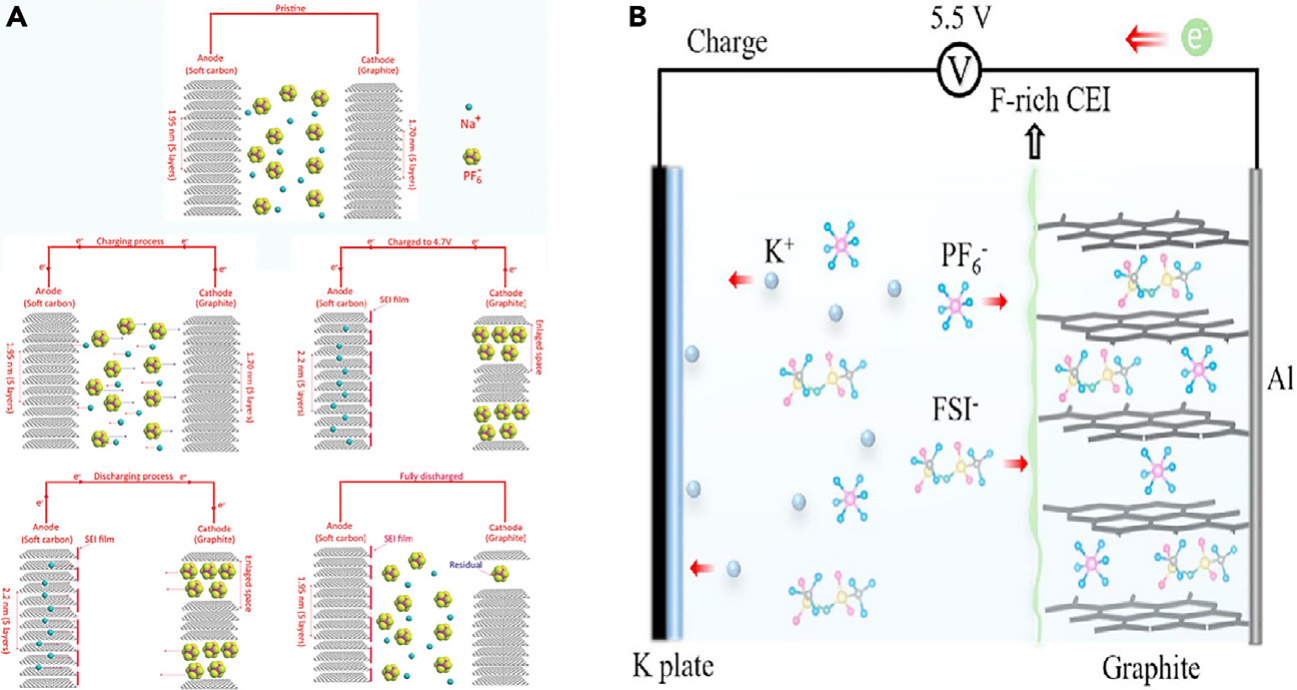
Schemes of DIBs (A) The schematic diagram of the DIBs using NaPF_6_ salt at the states of pristine, charging process, fully charged, discharging process, and fully discharged. Reprinted with permission from Fan et al.^106^ Copyright 2017 WILEY-VCH Verlag GmbH & Co. KGaA, Weinheim. (B) Scheme of K-based DIB. Reprinted with permission from Tan et al.^107^ Copyright 2021 Elsevier Ltd.

In 2018, Jiang et al.^105^ employed graphite as the positive electrode, hard carbon as the negative electrode, and combined them with a non-flammable trimethyl phosphate (TMP) solvent and a sodium bis(trifluoromethanesulfonyl)imide (NaTFSI) salt to construct a high-voltage and safer Na-based DIBs. This kind of electrolyte was beneficial to form a stable CEI layer on the cathode and achieved higher battery performance. The coordination between TMP/TFSI^−^ and Na^+^ effectively stabilized electron pairs and improved the oxidative stability of the solvent and anion. This mechanism was clearly demonstrated through DFT-MD simulations. For a NaTFSI:TMP ratio of 1:4, it highlighted the relatively scarce coordination of TMP, resulting in dominant free TMP molecules in the system (Figure 7A). However, in a NaTFSI:TMP ratio of 1:2 (Figure 7B), an appropriate NaTFSI to TMP molar ratio reduced the number of free solvents, effectively suppressing electrolyte decomposition. It showed a lower LDOS for TMP in the 1:2 NaTFSI:TMP electrolyte (Figures 7C and 7D), indicating that TFSI^−^ anions, with lower energy levels than TMP solvents, favorably reduce subsequent TMP decomposition on the electrode surface, forming a stable CEI layer. Additionally, Fan et al.^106^ reported a battery system, where soft carbon serves as the negative electrode, graphite as the positive electrode, and the electrolyte contains NaPF_6_ salt. They proposed the following mechanism for DIBs (Figure 8A): during the charging process, the applied voltage creates a potential difference, driving Na^+^ and PF_6_^−^ ions toward the soft carbon and graphite, respectively. With increasing voltage, the intercalation of Na^+^ and PF_6_^−^ gradually occurs in the soft carbon and graphite; After completing the charging process, Na^+^ and PF_6_^−^ have inserted into the soft carbon and graphite, respectively. The introduction of NaPF_6_ salt has profound effects on battery performance, especially regarding critical characteristics such as the formation of the CEI. The presence of NaPF_6_ salt enhances the electrolyte’s conductivity, facilitating ion transport within the battery and contributing to overall battery performance. NaPF_6_ salt may influence the solubility and chemical properties of the electrolyte, affecting the composition of CEI and leading to dynamic changes in CEI.

Recently, there has been significant attention on calcium (Ca) and potassium (K) based DIBs. For instance, Wu et al.^108^ have reported a high-performance calcium-ion DIB, employing environmentally friendly and cost-effective materials such as mesocarbon microbead (MCMB) as the negative electrode and expanded graphite as the positive electrode, featuring a dual carbon configuration design. The Ca-DIB demonstrated superior performance by using the electrolyte of Ca(PF_6_)_2_ in conventional carbonate solvent. During the charging process, Ca^2+^ ions migrate to the MCMB negative electrode, intecalating into the graphite layer to form CaC_x_ intercalation compounds. Simultaneously, PF_6_^−^ anions move to the expanded graphite positive electrode, intercalating into the graphite layer to form C_y_(PF_6_) intercalation compounds. After 10 cycles, a layer of CEI forms on the MCMB cathode, resulting in an increase in impedance compared to the first cycle. However, after 30 cycles, the impedance of the battery remains relatively unchanged, indicating that the CEI formed on the cathode is highly stable. Although CEI undergoes continuous dissolution and regeneration processes during battery cycling, the newly formed CEI does not impose significant resistance to anion migration. It remains thin and uniform, providing excellent pathways for anion intercalation into graphite, ensuring smooth transport. Tan et al.^107^ adopted a dual anions intercalation strategy to enhance the performance of K-based DIBs. In comparison to the widely used single anion intercalation methods, this dual anion intercalation strategy demonstrated significant advantages in achieving high capacity and stable graphite-based positive electrodes. To investigate the chemical stability, surface analysis of pure graphite powder cycled with dual-ion and single salt electrolytes was conducted using XPS. It presented the C1s spectrum of 0.8 M KFSI, revealing the formation of C-O, C=O, and C-F substances (Figure 7E).^109^^,^^110^^,^^111^ Meanwhile, in 0.3 KFSI-0.5 KPF_6_ and 0.7 M KPF_6_ electrolytes, the graphite surface significantly enriched in C-F substances, along with C-F_2_ and C-F_3_ substances. Thus, the F1s spectrum confirmed the presence of a small amount of CHF-CH_2_, CH_2_-CF_2_, and CF_2_-CF_2_ substances on the graphite surface in 0.8 M KFSI electrolyte (Figure 7F).^112^^,^^113^^,^^114^ The fluorine in the resulting CEI mainly originates from the decomposition of PF_6_^−^ under high voltage. It has been demonstrated that the generation of fluorine-rich substances is beneficial for stabilizing the CEI under high pressure, explaining how the dual anion electrolyte achieves excellent oxidative stability at high potentials and enhances battery performance (Figure 8B).

Besides, high-concentration electrolytes (HCE) and low-concentration electrolytes (LCE) will have different effects on the formation of CEI layers on the graphite surface. HCE can provide a higher ion concentration, reducing the concentration of free solvent molecules by increasing the content of anions. Therefore, controlling different interface reactions on the electrode surface can be achieved by altering the solvation structure of ions, aiding in the formation of a more stable CEI layer.^89^^,^^115^^,^^116^^,^^117^ However, the state-of-the-art development of using HCE still faces some challenges. Most of these studies are based on carbonate solvents with relatively low oxidative stability, leading to the continuous decomposition of solvents at the electrode-electrolyte interface.^98^^,^^118^ Moreover, to avoid electrolyte decomposition, the upper voltage limit for DIBs is usually kept below 5 V, which is detrimental to improving energy storage performance.^71^^,^^119^ However, there is scarce literature reporting the fundamental mechanisms behind performance enhancements.^120^ Therefore, further optimization of the electrolyte system is needed to address the aforementioned challenges of DIBs from CEI perspective.

**Figure 9:**
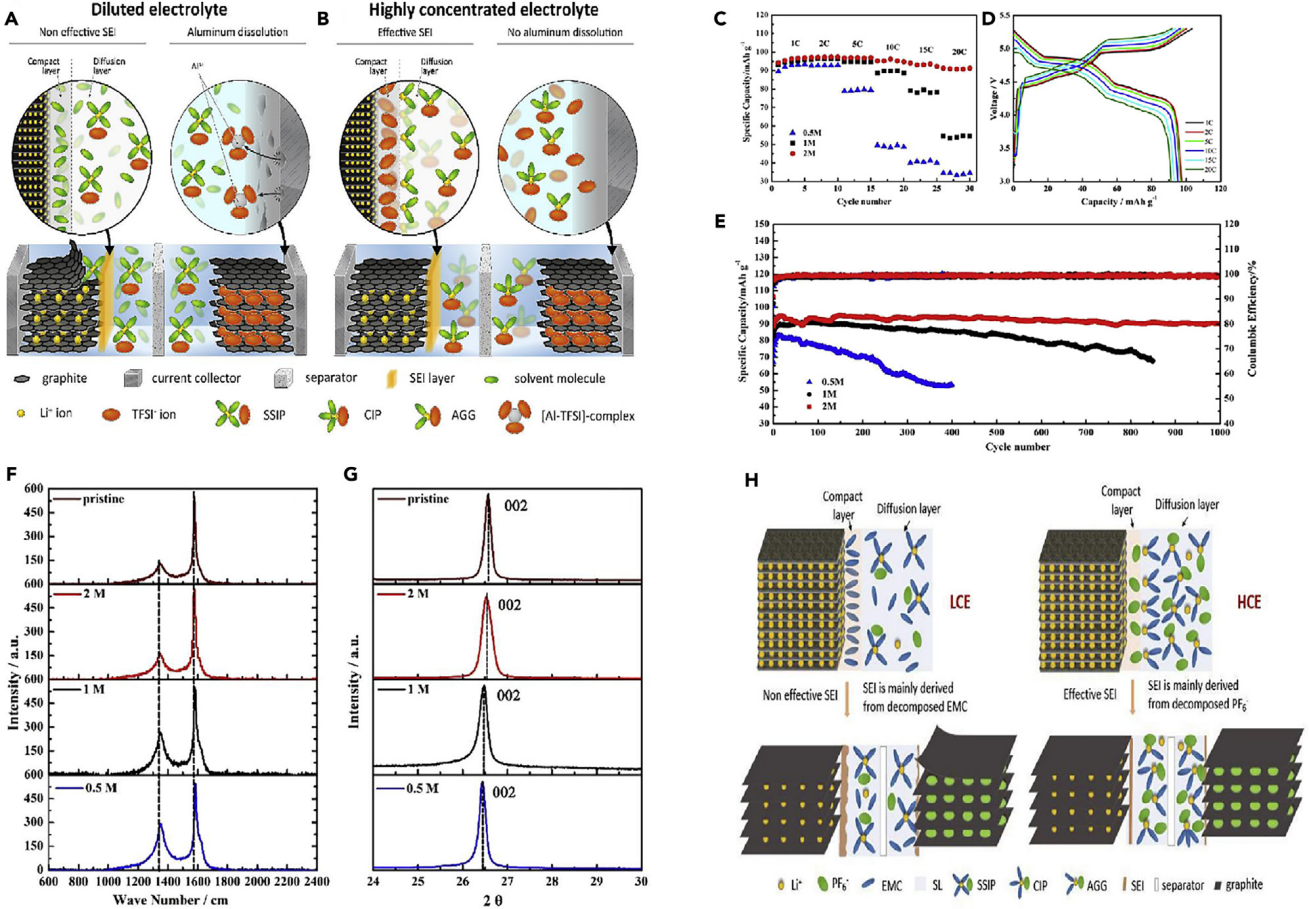
Schemes of mechanism and battery performances (A and B) Working mechanisms and (dis-)advantages of the dual-graphite battery using (A) diluted and (B) highly concentrated electrolytes; SSIP, a solvent-separated ion pair; CIP, contact ion pair; AGG, aggregated ions. Reprinted with permission from Heckmann et al.^115^ Copyright 2017 Elsevier Ltd. (C) Rate performance of the three DGBs. (D) Galvanostatic charge-discharge profiles of the DGB in 2 M electrolyte. (E) Cycle performances of the DGBs at 5 C. (F) Raman spectra and (G) XRD patterns of the pristine graphite and cycled graphite cathodes in DGBs using 0.5, 1.0, and 2.0 M electrolytes. (H) The formation mechanisms of the SEI and CEI in DGBs using dilute electrolytes and HCE. Reprinted with permission from Liu et al.^120^ Copyright 2019 Elsevier B.V.

Heckmann et al.^115^ investigated the application of LiTFSI-based and LiPF_6_-based HCE in DIBs. HCE is appealing for use in DIBs, particularly on graphite, due to its ability to create a functional SEI,^121^ significantly improving the reduction stability and the reversibility of Li^+^ intercalation/de-intercalation. This effect is generated by the synergistic action of salt and solvent molecules without the need for electrolyte components containing EC.^122^^,^^123^ Furthermore, HCE can also enhance the stability of the oxidation electrolyte on the positive electrode while suppressing the dissolution of aluminum in the salt-solvent combination (Figure 9A). These combinations in LCE do not form a protective fluoride layer on the surface of aluminum, as seen in acyl imide-based salt electrolyte formulations.^124^^,^^125^ The use of HCE can enhance battery safety by reducing solvent vapor pressure and enhancing thermal stability.^125^ However, using HCE also has some disadvantages, such as increased demand for electroactive salt, leading to higher electrolyte costs, and typically reduced ionic conductivity. Nevertheless, the advantages in electrochemical performance may outweigh these drawbacks. In the case of diluted electrolytes, inappropriate components may lead to rapid battery failure due to graphite exfoliation or extensive aluminum dissolution (Figure 9B), whereas HCE can mitigate these issues. Liu et al.^120^ found that due to the increased proportion of contact ion pairs and ion aggregates, HCE can suppress structural collapse of the graphite positive electrode. This will facilitate the development of DIBs with high-rate performance and excellent cycling stability. Moreover, HCE (2 M) can enhance rate performance compared to LCE (0.5 M), but with reduced ionic conductivity (Figure 9C). The DIBs exhibited significantly enhanced rate capability in HCE, attributed to improved compatibility of the negative electrode-electrolyte interface, resulting in a uniform SEI on anode with highly reversible Li^+^ intercalation/de-intercalation characteristics.^126^ Furthermore, the GDIB cycled in HCE exhibited much lower polarization compared to those cycled in diluted electrolytes (Figure 9D). It demonstrated a significant improvement in the cycling performance of GDIBs with increasing electrolyte concentration (Figure 9E). Changes in the crystallinity of cycled graphite positive electrodes in different electrolytes were analyzed by Raman and XRD measurements. Graphite positive electrodes cycled in HCE maintained a highly integrated graphite structure compared to diluted electrolytes (Figures 9F and 9G). Raman and XRD results indicated that HCE alleviates structural collapse of the graphite positive electrode. Zheng et al.^123^ reported that the formation mechanism and properties of the CEI layer formed in HCE and diluted electrolytes are significantly different. The composition of the CEI layer includes a dense layer and a diffusion layer. In diluted electrolytes, the formation of the dense layer is mainly determined by free solvent molecules, thus the CEI layer primarily originates from the decomposition of free solvent molecules (Figure 9H).

In summary, using HCE and LCE will have different effects on the formation of CEI on graphite surfaces. HCE can increase the thickness of the CEI layer, improving battery cycling life but may raise safety concerns. LCE can enhance battery power density and safety but may accelerate capacity decay. Therefore, when selecting electrolyte concentration, the performance and safety requirements of the battery need to be considered comprehensively.

#### Main solvents

Solvents provide a medium that allows ions to move freely in the electrolyte. The molecular structure of solvents typically surrounds ions, enabling their conduction in the electrolyte, thereby facilitating the formation of a CEI layer on the graphite cathode during the charge-discharge processes of a battery. Organic solvents, not only exhibit higher stability under high pressure but also contribute to the formation of a robust CEI during interfacial reactions. In DIBs, commonly employed organic carbonate solvents include EC, propylene carbonate (PC), EMC, dimethyl carbonate (DMC), and diethyl carbonate (DEC). These solvents offer advantages such as low cost, high ionic conductivity, high dielectric constant, low viscosity, and an appropriate electrochemical stability window.^29^^,^^93^^,^^102^^,^^115^^,^^127^^,^^128^

**Figure 10:**
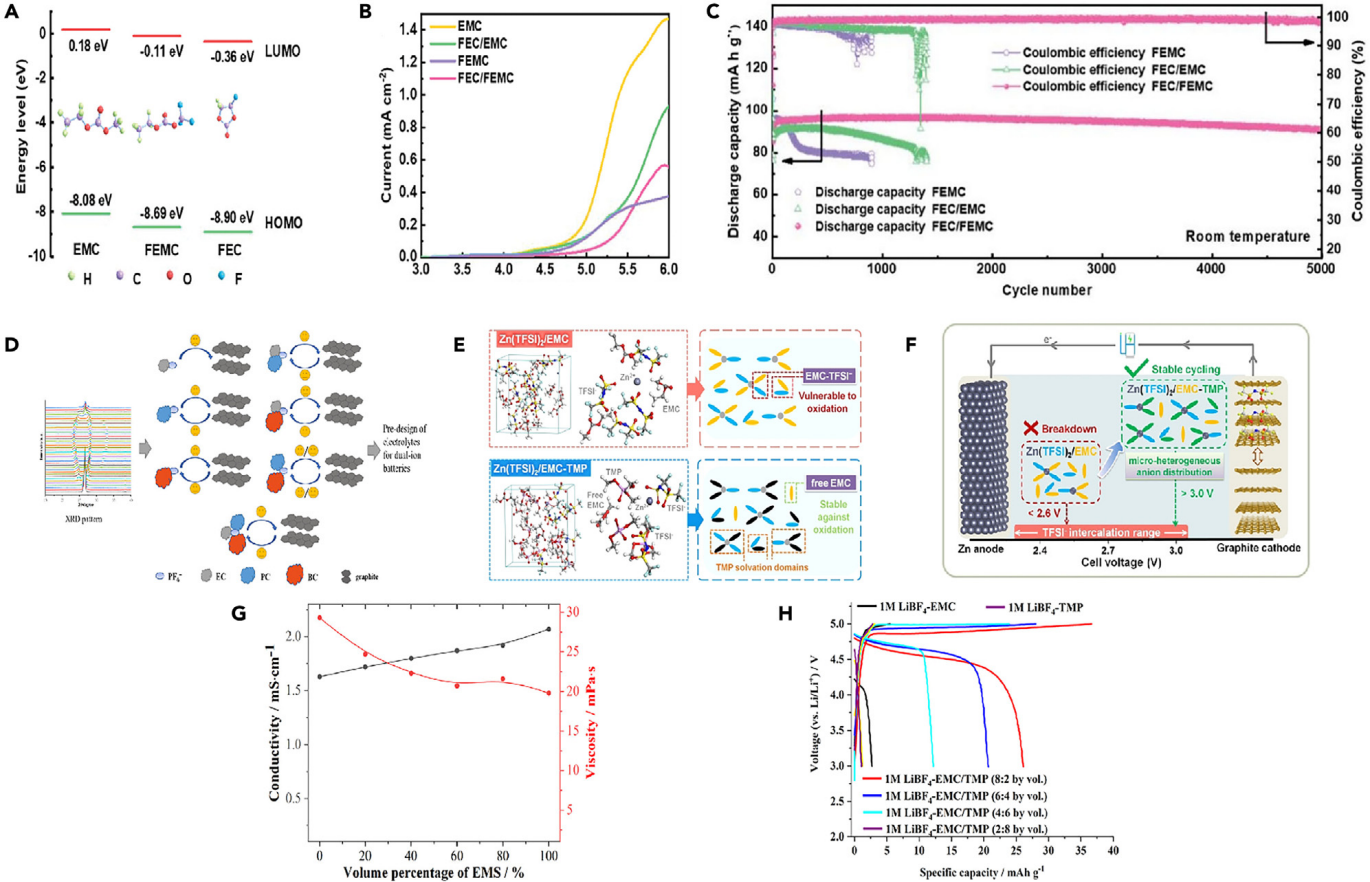
Calculations, scheme and battery performances (A) Structures and molecular orbital energies of EMC, FEMC, and FEC solvents. (B) LSV curves of a titanium electrode in 1 M LiPF_6_-based EMC, FEC/EMC, FEMC, and FEC/FEMC electrolytes at a scan rate of 5 mV s^−1^. (C) Cycle performance and Coulombic efficiency of graphite||Li cells. Reprinted with permission from Wang et al.^56^ Copyright 2022 Wiley-VCH GmbH. (D) Schematic representation of the performance and solvation state of graphite cathode dissolved in 1 M LiPF_6_ solution of conventional cyclic carbonates including EC, PC, BC, and their mixtures. Reprinted with permission from Wang et al.^132^ Copyright 2021 American Chemical Society. (E) AIMD results of the solvation structure: (E) Snapshots of the simulation cells and representative solvation structures for 1.5 M Zn(TFSI)_2_/EMC and 1.5 M Zn(TFSI)_2_/EMC-TMP (1:3 by volume) (Zn, violet; O, red; N, blue; C, gray; S, yellow; F, cyan; H, white). (F) Design strategy of EMC-based electrolyte with a micro-heterogeneous anion solvation network for high-voltage Zn/graphite cells. Reprinted with permission from Chen et al.^129^ Copyright 2020 Wiley-VCH GmbH. (G) Conductivity and viscosity of 1 M LiBF_4_-EMS/SL solutions. Reprinted with permission from Wang et al.^133^ Copyright 2022 American Chemical Society. (H) Initial galvanostatic charge/discharge curves of graphite electrodes in the electrolyte solutions of 1 M LiBF_4_ EMC/TMP. Reprinted with permission from Zhang et al.^134^ Copyright 2019 American Chemical Society.

In 2022, Wang et al.^56^ achieved significant progress once again by adding FEC as a co-solvent. DFT simulations indicated that among the three solvents, FEC exhibited the highest oxidation stability, while EMC had the highest oxidation tendency at high voltages. Introducing strong electron-withdrawing groups (such as -CF_3_) into the solvent structure significantly lowered the HOMO and LUMO energy levels, thereby enhancing the oxidation and reduction potentials of the solvent (Figure 10A). FEC and FEMC effectively expanded the electrochemical stability window to 5.5 V, which was consistent with the DFT calculation results (Figure 10B). The use of a fully fluorinated electrolyte as an innovative measure resulted in the formation of a dense CEI layer on the graphite cathode surface, successfully suppressing parasitic side reactions and significantly improving the cycling stability of graphite||Li batteries. Additionally, the adoption of fluorinated electrolytes not only improved the cycling stability of the batteries (Figure 10C), but also demonstrated excellent cycling stability, indicating that replacing EMC with FEMC to suppress parasitic reactions, attributed to the stable CEI layer, effectively inhibited electrolyte decomposition and self-discharge. They conducted a performance comparison of batteries at room temperature and at 0°C. A DIB using 1 M LiPF_6_-FEC/FEMC maintained 97.8% of its available capacity and exhibited excellent voltage profile and cycling stability at both temperatures. Even after cycling 3000 times at a high current rate of 5 C, the capacity did not degrade. These results indicate that for DIB, this perfluorinated electrolyte enables rapid and reversible reaction kinetics between PF_6_^−^ and lithium at low temperatures. In rocking-chair batteries such as LIBs, the ion solvation energy in the electrolyte plays a crucial role in electrode reaction kinetics, as solvent molecules must be desolvated from lithium solvation structures near the negative and positive electrodes during charging and discharging. Therefore, slow desolvation of lithium can affect the charging and discharging performance of batteries, especially at low temperatures. Furthermore, according to previous studies,^129^^,^^130^ the interaction between anions and solvent molecules is much weaker, leading to the rapid desolvation of anions and thus enhancing rate and low-temperature performance. Wang et al.^28^ conducted more detailed measurements on 1 M LiPF_6_-MA/DEC (A8E2) to explore its potential, which could be amplified even at low temperatures. The charge-discharge curves of lithium-ion/graphite batteries based on A8E2 showed minimal variation at different temperatures of 20°C and −20°C, compared to the significant voltage curve degradation of traditional lithium-ion batteries at low temperatures. This suggested that batteries using A8E2 can maintain good performance at low temperatures, partly due to its high ionic conductivity in solution (9.29 mS cm^−1^ at −20°C). Battery cycling capabilities using A8E2 were satisfactory at both room temperature and low temperature. After cycling 1000 times at 5 C at 20°C, the battery capacity remained at 85.35% of the initial capacity. Moreover, the long-term cycling performance at low temperatures was close to that at room temperature, with a capacity retention rate of 83.24% after cycling over 1000 times at −20°C. While the rate capability of batteries using A8E2 decreased somewhat at low temperatures, this trend was not significant compared to other solutions, indicating the formation of a stable CEI film at low temperatures. Therefore, a certain energy and power density can still be achieved at low temperatures. What’s even more impressive is that the battery containing the A8E2 can operate at minus 70°C. The A8E2 can also be used in a variety of other applications. He et al.^131^ found that adding FEC solvent did not result in a distinguishable CEI layer on the graphite cathode surface. According to electron energy loss spectroscopy (EELS) and XPS characterization, only scattered by-products could be detected. Furthermore, the electrolyte with FEC solvent indeed helped alleviate graphite exfoliation and enhance the stability of the cathode. The by-products on the cathode were dispersed rather than uniformly distributed on the graphite surface. In LiPF_6_ EMC/FEC, the proportion of LiF in the by-products significantly decreased, indicating that the decomposition of LiPF_6_ in the mixed solvent was inhibited. The influence of FEC was not as typically stated in the literature as regulating the composition of the CEI. It is more reasonable to consider the FEC effect as weakening the dissolution effect. Local lattice disorder and layer bending were also observed in the cycled graphite cathode, attributed to local oxidation under high working voltage and high lattice strain caused by anion intercalation. This study further indicated that the absence of the CEI layer was the fundamental reason for the rapid self-discharge of the graphite cathode.

Carbonates are widely used in the electrolytes of DIBs due to their various properties such as dielectric constant, dynamic viscosity, density, and electrochemical stability. These properties depend on the chemical structure and composition of carbonates. Ring-shaped carbonates (such as EC and PC) are common examples, possessing higher dielectric constants that aid in the dissolution of electrolyte salts. In contrast, linear carbonates (such as EMC, DMC, and DEC)^99^^,^^135^^,^^136^^,^^137^ reduce viscosity and enhance ion conductivity.^138^^,^^139^

Wang et al.^132^ investigated the performance and dissolution state of graphite cathode dissolved in 1 M LiPF_6_ solutions of conventional cyclic carbonates including EC, PC, butylene carbonate (BC) and their mixtures. The storage behavior of PF_6_^−^ in graphite electrodes was elucidated by several electrochemical experiments combined with *in situ* XRD measurements. The intercalation of more than two carbonate co-soluble anions in graphite was found. It was also found that the storage performance of PF_6_^−^ in graphite electrodes was closely related to the composition of the electrolyte solution (Figure 10D). Selection of the solvent can affect the stability of the CEI layer and the cycle life of the cell. Chen et al.^129^ performed ab initio molecular dynamics (AIMD) simulations in which a large number of intimate EMC-TFSI^−^ existed in the pristine Zn(TFSI)_2_ in EMC solution, which were readily oxidized by the anionic nucleophilic attack on the EMC at high potentials, and when TMP was introduced as a structural disrupter in the solution, the TFSI^−^ anions are disconnected from the EMC and confined to the TMP solvation in the form of TMP-TFSI^−^ complexes and TMP-solubilized Zn^2+^ participating in the ion pair. As a result, the EMC, which was originally complexed with the anion, can be released to a free state, thus regaining its intrinsic tolerance to oxidation (Figure 10E). A microheterogeneous anionic solvation structure was formed in which the TFSI^−^ anion is confined to the TMP-rich structural domains and the affinity of the anion for the carbonate was greatly reduced (Figure 10F). Improved compatibility of the components in the electrolyte and formation of a stable CEI. It was also confirmed that the EMC-based electrolyte could form a thin layer of CEI on the graphite surface with fewer Li-F and ROCO_2_Li species compared to DMC and DEC, which effectively reduces the charge-transfer resistance, polarization, and self-discharge of the graphite electrodes, resulting in improved cathode-electrolyte interfaces, cycling, and rate performance of the DIB.^29^ The solvent has an effect on the anion intercalated in graphite and therefore determines the electrochemical performance of the DIBs to some extent. Since the interaction between the solvent and ions in the solution can be reflected from the ionic conductivity, Wang et al.^133^ compared the ionic conductivity values of 1 M LiBF_4_ EMS/SL solutions (Figure 10G). The conductivity increased steadily with increasing EMS content. This trend can be attributed to the lower viscosity and higher dielectric constant of EMS. Optimization of the anionic solvation structure of the electrolyte can improve the ion transport performance in the CEI layer and increase the migration rate, thus enhancing the charging and discharging performance and power density of the battery.

To prepare concentrated electrolytes, a combination of high dielectric constant carbonates (such as EC) and low viscosity carbonates (such as DMC and EMC) can be mixed in specific proportions.^140^^,^^141^ This combination achieves a balance between ionic conductivity and salt concentration, thus meeting the demands for both high power and high energy performance. This mixing strategy not only considers the electrochemical properties of carbonates but also takes into account their physical properties, such as viscosity and density. Such a balanced combination helps improve the overall performance of batteries, making them suitable for various applications. Recently, Ji et al.^135^ recently succeeded in developing an innovative electrolyte with 1 M KPF_6_ added to the EC/DMC/EMC solvent. where the EC content is relatively high, the high EC content improves the solubility of KPF_6_, improves the solvated structure of the electrolyte, increases the ionic conductivity, and the mixed solvent can form a stable solvated shell, and the ionic pairs of free ions in the electrolyte are stabilized. It could suppress the decomposition of the electrolyte under high voltage, and a stabilized CEI formed on the expanded graphite positive electrode positively affects the stability and cycle life of the electrode.

Furthermore, Tong et al.^142^ successfully dissolved 4 M LiFSI salt in tetramethylene sulfone (TMS) solvent, constructing an innovative electrolyte system. This system formed a stable and effective CEI layer on both positive electrodes, achieving a high oxidation potential (approximately 6.0 V), thus enabling reversible FSI^−^ intercalation/de-intercalation in the graphite cathode. This design significantly suppresses gas generation at high operating voltages and significantly improves the energy density of the full-cell DIBs. Zhang et al.^134^ designed a 1 M LiBF_4_ EMC/TMP hybrid electrolyte solution (8:2 volume ratio) has been used for lithium/graphite DIBs, and its capacity performance is far superior to that of an electrolyte solution with 1 M LiBF_4_ dissolved in EMC or TMP pure solvent (Figure 10H). By designing a good electrolyte environment with excellent oxidative stability of the cathode, the CEI formed was more stable and efficient in passing anions, improving battery performance.

Optimizing the electrolyte for DIBs has various impacts on the CEI layer. Selecting the concentration and type of ions in the optimized electrolyte can improve ion transport properties within the CEI, reducing diffusion resistance, enhancing migration rates, and consequently improving the capacity and power density. An appropriate solvent selection can affect the stability of the CEI and the cycling life of the battery. Choosing stable, non-volatile, and well-dissolving solvents can reduce solvent precipitation and solution concentration effects, delaying the rate of CEI formation and enhancing battery stability and lifespan. Adjusting the type and concentration of additives in the electrolyte can control the formation and properties of the CEI layer. For example, adding appropriate amounts of surfactants can improve the wetting between the electrolyte and electrodes, promoting ion transport and reactions. Simultaneously, adding inhibitors can prevent electrolyte decomposition and electrode surface degradation, enhancing battery stability and durability. It is important to note that the influence of optimized electrolytes on the CEI layer is a complex issue that requires the comprehensive consideration of factors such as the compatibility, electrochemical stability, and ion transport properties of the components in the electrolyte. Therefore, for specific DIB systems, the optimal electrolyte formulation and conditions need to be determined through experimentation and evaluation to meet the performance requirements of the battery.

### Artificial cathode electrolyte interphase

The artificially manufactured CEI in DIBs has multifaceted significance. As the interface layer between the electrolyte and positive electrodes of the battery, the CEI achieves precise control over electrochemical reactions by regulating its surface chemical properties. By artificially manufacturing the CEI, penetration of solvents and ions can be restricted, and the safety of the battery by preventing electrolyte leakage and electrode short circuits can be also enhanced, reducing the risk of combustion and explosion.^27^^,^^48^^,^^83^^,^^143^^,^^144^

**Figure 11:**
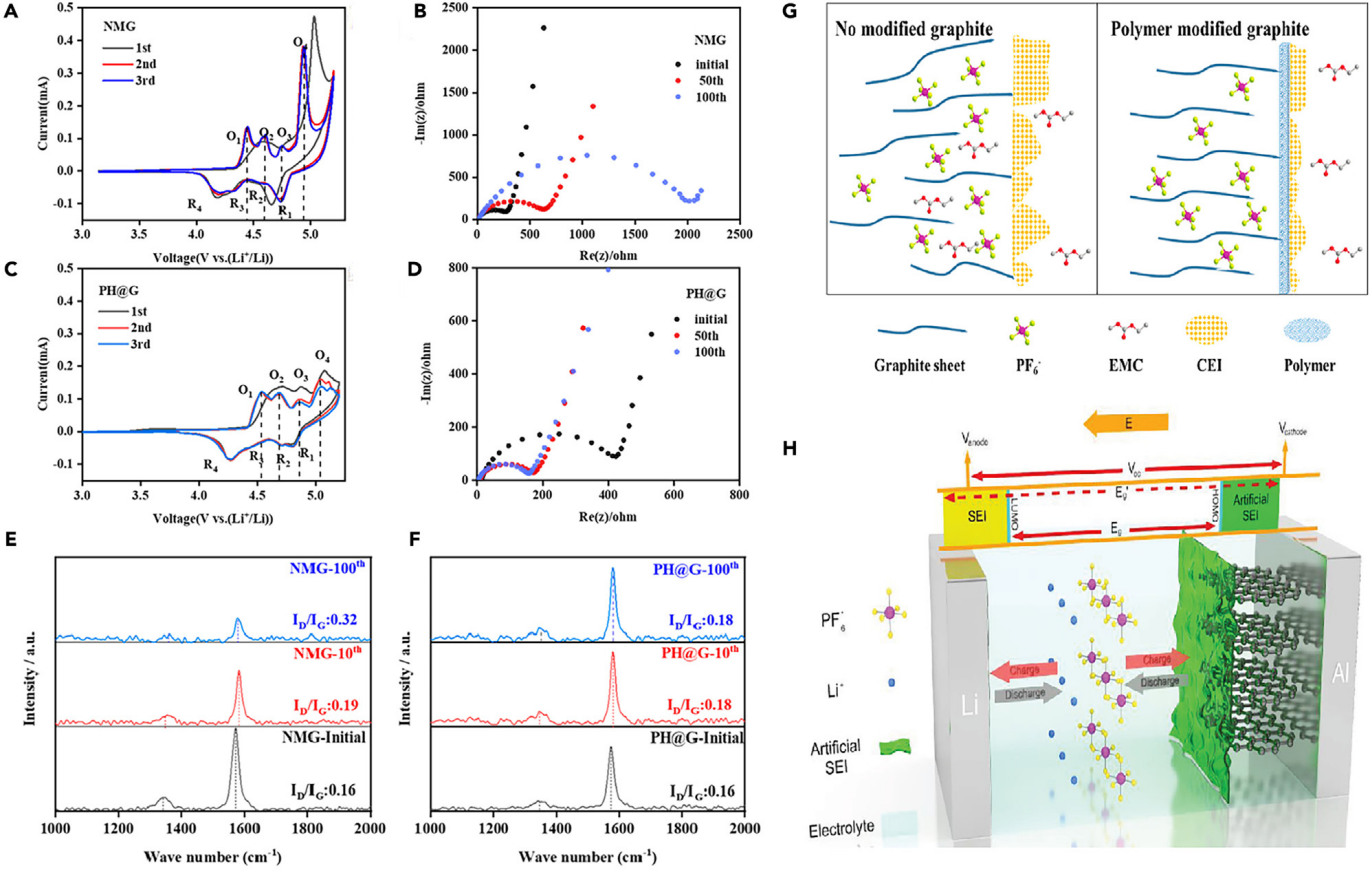
Electrochemical results and schemes (A–D) CV curves and EIS impedances of (A, B) NMG and (C, D) PH@G. (E and F) Raman spectra of (E) NMG and (F) PH@G (initial, after 10 cycles, and after 100 cycles). (G) Illustration of the protection mechanism of artificial polymer CEI on PF_6_^−^ intercalated graphite. Reprinted with permission from Liu et al.^145^ Copyright 2023 American Chemical Society. (H) Scheme of the working mechanism of Li-based DIB with artificial CEI on graphite cathode. Reprinted with permission from Li et al.^89^ Copyright 2018 WILEY-VCH Verlag GmbH & Co. KGaA, Weinheim.

Recently, Liu et al.^145^ have successfully developed a revolutionary technology to improve the artificial PVDF-HFP polymer CEI layer on the graphite cathode. This innovative technology significantly inhibits electrolyte decomposition, elevates the Coulombic efficiency level, and maintains the stability of the graphite structure during the (de-)intercalation process (Figure 11G). They proposed that in DIBs, initially, the active carriers are stored in the electrolyte; during charging, anions migrate to the positive electrode, undergo electrochemical conversion, and are stored in the positive electrode material, simultaneously releasing electrons flowing to the negative electrode. Meanwhile, cations migrate to the negative electrode and react with the negative electrode material to accept electrons. Ideally, the number of electrons released by the positive electrode storing anions equals the number of electrons needed by the negative electrode to accept cations. If there is strong irreversible electrolyte oxidation at the positive electrode, cations may accumulate in the negative electrode as charging progresses, reducing the available storage capacity, and resulting in CE below 100%. It has been reported that surface CEI modification strategies can prevent electrolyte decomposition, and artificial CEI protectants enhance the stability window of the electrolyte to a broader range. As expected, the irreversible stripping of the graphite electrode is significantly reduced, and CE is improved.^89^ Particularly, when storing anions at high voltage, side reactions at the positive electrode are more likely to occur. Compared with the CE of the two samples within 180 cycles, at a cutoff voltage of 5.2 V, the CE of artificially modified cathode (PH@G) is higher than that of batteries without artificial modification (NMG), indicating that the artificial PVDF-HFP CEI reduces side reactions at the positive electrode. Generally, except for the first cycle, the CV curves of the two samples overlap well in each cycle; compared to NMG, the CV curves of PH@G exhibit distinct oxidation peaks above 5.0 V, indicating different side reactions occurring at the PH@G electrode surface (Figures 11A and 11B**)**. EIS measurements were conducted for further investigation (Figures 11C and 11D**)**. The charge transfer resistance (R_CT_) of NMG is significantly lower than that of PH@G in the initial cycles, suggesting an increase in anion transfer resistance at the graphite surface polymer interface. After 50 cycles, the R_CT_ scan of PH@G is lower than NMG. Furthermore, the R_CT_ value of PH@G after 50 cycles is significantly lower than the initial cycle, indicating that the charging process within 50 cycles perfectly alters the structure of the PVDF-HFP interface, rendering the interface with improved electrochemical performance. Previous reports have indicated that the structural changes in graphite are directly correlated with the energy storage mechanism and stability of LIBs.^118^ Raman spectroscopy can be utilized to analyze the crystalline changes in graphite after cycling. The peaks at 1580 cm^−1^ (G band) and 1350 cm^−1^ (D band) respectively represent the lattice and disordered structures (such as defects) in graphite.^146^^,^^147^ The extent of defects in graphite can be estimated by the intensity ratio of the D band to the G band (I_D_/I_G_). After 10 and 100 cycles, the I_D_/I_G_ of NMG increased from 0.16 to 0.19 and 0.32, respectively. These results suggest that the layer structure of graphite gradually changes after repeated PF_6_^−^ de/intercalation processes (Figures 11E and 11F**)**. In contrast, the I_D_/I_G_ of PH@G slightly increased, indicating that the PVDF-HFP assisted in constructing the CEI, which can suppress harmful collapse of the graphite layer structure. Both XRD and Raman results indicate that the artificial CEI on graphite cathodes can maintain the graphite structure after cycling by reducing solvent co-intercalation, resulting in a more ordered and highly dense structure, greatly enhancing the cycling stability of the PF_6_^−^ de/intercalation process. The crystal structure of NMG gradually collapses during the PF_6_^−^ de/intercalation cycling process. Li et al.^89^ successfully generated an artificial CEI on modified graphite electrodes over five cycles, which significantly enhanced the cycling performance of lithium-graphite batteries (Figure 11H**)**. They conducted a series of galvanostatic charging/discharging cycles at a rate of 100 mA g^−1^ within the potential window of 0.3–2.0 V. The CEI protective layer not only effectively suppressed the decomposition of the positive electrode electrolyte but also alleviated the solvent solvation effect of anions within the battery, ensuring the stability of the graphite surface region.

## Other strategies

In addition to liquid-solid electrolyte engineering and utilization, there are many different methods to stabilize the CEI, such as element doping and graphite cathode structure design, and these improvements have achieved gratifying results.

### Dopant element

DIBs are an emerging ion storage technology, and many people have been exploring various methods to improve their performance.^53^^,^^148^^,^^149^ Among them, the influence of doping elements on the CEI is an important research direction. Doping elements can introduce different atoms into the graphite structure, altering the physical and chemical properties of graphite, thereby affecting the formation and stability of the CEI layer. Fluorine doping: Fluorine doping can improve the electrochemical stability of the CEI layer.^97^ Nitrogen doping: Nitrogen doping can increase functional groups on the graphite surface, improving compatibility between the CEI layer and electrolyte.^150^ The introduction of both fluorine and nitrogen atoms can reduce oxidation reactions and solvent decomposition on the graphite surface, thereby inhibiting the continuous growth and thinning of the CEI layer, and enhancing its stability. In addition to the above doping elements, other doping elements such as phosphorus,^151^ oxygen,^152^ and so forth, have also been studied to improve the performance of the CEI layer. However, the selection and optimization of doping elements still require further research and exploration.

Boron doping can slow down the reaction between graphite and solvents in the electrolyte, reduce the solvent decomposition and deposition of electrolyte salts, thereby lowering the resistance of the CEI layer.^153^^,^^154^^,^^155^ Recently, Zhang et al.^154^ proposed a concise and innovative design scheme for constructing positive and negative electrode materials to achieve efficient transport of PF_6_^−^. By adjusting the doping amount of boron based on coordination chemistry during the synthesis process, a three-dimensional pore structure was successfully introduced, providing sufficient space for anion intercalation, effectively shortening the anion volume phase transfer path. Boron doping in cathodes enhances the initial Coulombic efficiency and stability of the CEI by suppressing electrolyte decomposition, as evidenced by the higher initial Coulombic efficiency from 48% to 55%, and reversible capacity retention over cycles.

### Graphite cathode structure design

There are other modification methods of the graphite cathode surface which can improve the formation and performance of the CEI layer in DIBs. Firstly, chemical modification introduces chemical substances such as oxidation and silicification to alter the chemical properties of the graphite cathode surface.^97^^,^^156^^,^^157^ For example, oxidation treatment introduces functional groups to enhance interaction with the electrolyte,^158^ thereby aiding in improving the formation and stability of the CEI layer. Secondly, surface coating utilizes materials such as polymers and metal oxides to form thin films,^74^^,^^159^^,^^160^ providing a protective layer to prevent direct contact between substances in the electrolyte and the graphite cathode, reducing harmful reactions, and improving the stability of the CEI layer. Thirdly, nanomaterial modification introduces nanoparticles,^161^^,^^162^ nanosheets,^163^ and so forth, to provide more active surface area and enhance electron conduction pathways, thereby enhancing the anion intercalation capability and electrochemical performance of the graphite cathode.

**Figure 12:**
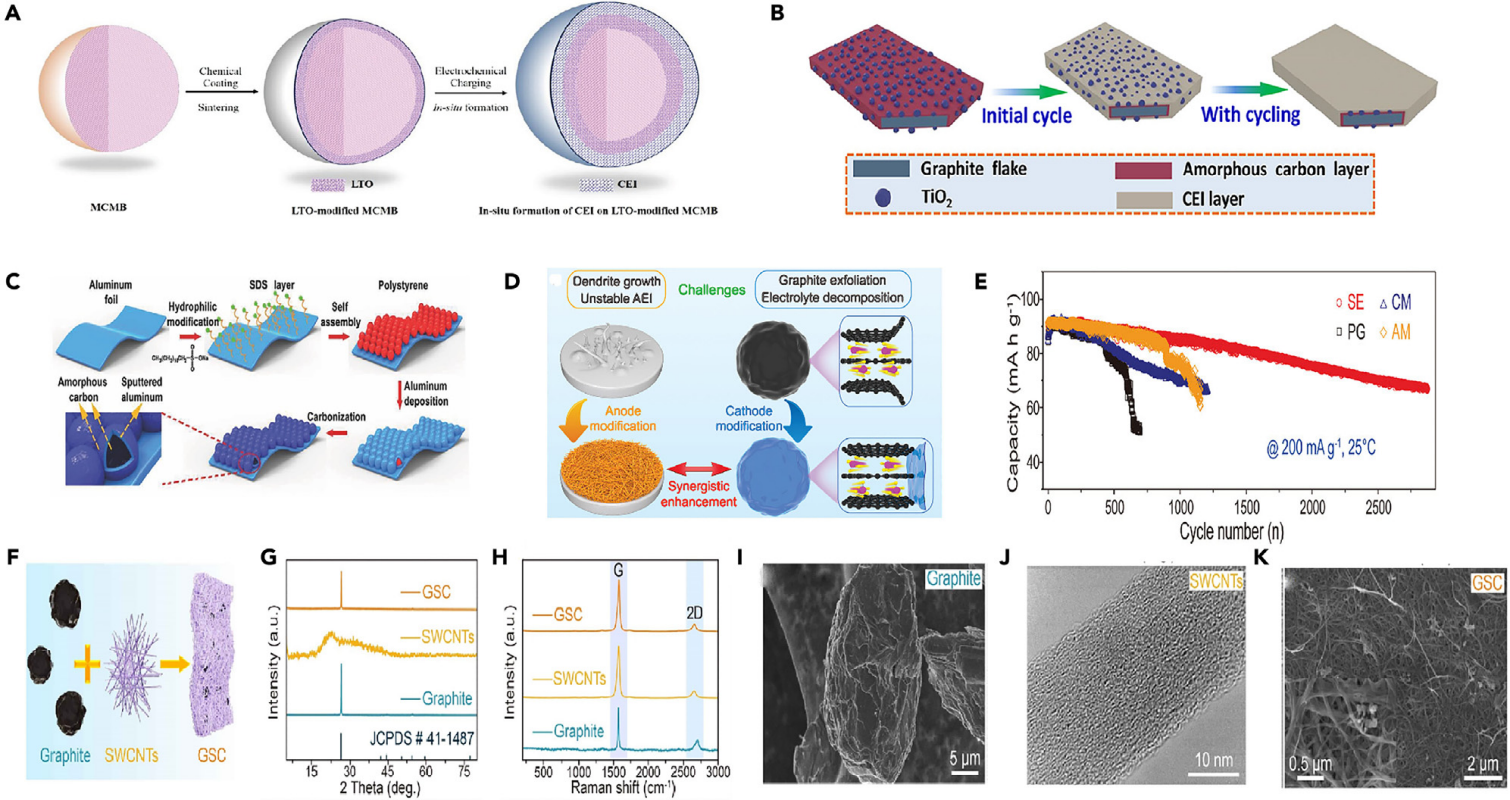
Preparation precedures and characterizations (A) Schematic diagram of the chemical coating and *in situ* polymerization of the CEI on LTO-modified MCMB. Reprinted with permission from Han et al.^159^ Copyright 2019 WILEY-VCH Verlag GmbH & Co. KGaA, Weinheim. (B) Illustration of the chemical modification CEI layer on TiO_2_/carbon co-modified graphite flake cathode. Reprinted with permission from Han et al.^164^ Copyright 2021 American Chemical Society. (C) Schematic illustration of the carbon-coated hollow aluminum nanospheres fabrication process for the DIB. Reprinted with permission from Qin et al.^165^ Copyright 2017 WILEY-VCH Verlag GmbH & Co. KGaA, Weinheim. (D) Schematic illustration of the main challenges in Li||Graphite DIBs, and the proposed two-pronged strategy to address them. (E) Typical cycling performance of PG, AM, CM, and SE at a current density of 200 mA g^−1^ and 25°C. Reprinted with permission from Li et al.^166^ Copyright 2022 Wiley-VCH GmbH. (F) Schematic diagram of the preparation of graphite-SWCNTs composite (GSC). (G) XRD patterns of graphite, SWCNTs, and GSC, and their corresponding (H) Raman spectra. (I) Typical SEM image of graphite. (J) Typical high-resolution TEM image of SWCNTs. (K) Typical SEM image of GSC. Reprinted with permission from Li et al.^87^ Copyright 2022 Wiley-VCH GmbH.

Recently, Han et al.^159^ proposed an innovative *in-situ* electrocatalytic strategy that combines material chemistry, electrocatalysis, and interfacial chemistry. In this strategy, the positive electrode of MCMB is chemically coated through a Li_4_Ti_5_O_12_ (LTO) layer, which not only serves as a framework but also provides electrocatalytic active sites, facilitating the formation of a favorable CEI layer (Figure 12A). Through the synergistic action of LTO and CEI networks, the thermodynamic behavior of PF_6_^−^ intercalation process can be altered while maintaining the structural integrity of the graphite positive electrode, forming highly stable graphite intercalation compounds. This synergistic LTO-CEI network acts as a “Great Wall” protecting the positive electrode at the interface, effectively preventing structural collapse and electrolyte decomposition. Furthermore, Han et al.^164^ reported an efficient nanostructure modification strategy achieving ultra-long cycling stability of 5 V graphite positive electrode. This strategy involved introducing nanostructured materials onto the graphite surface to form a stable CEI layer (Figure 12B). They synthesized a nano-material, namely TiO_2_/graphite flake (TO/GF), where TiO_2_ and carbon are uniformly distributed and tightly bound to the graphite flake surface. TO/GF exhibited remarkable stability during the charge/discharge cycling storing PF_6_^−^ anions. After 10,000 cycles, the positive electrode material demonstrated excellent performance, significantly surpassing other high-voltage positive electrode materials. They demonstrated through XRD spectroscopy that both the graphite flakes in the GF sample and TO/GF exhibit extremely high crystallinity. Higher graphitization levels result in enhanced electrochemical performance for anion storage. A minor diffraction peak observed at 2θ = 25.28° indicates the presence of titanium dioxide in TO/GF. Further characterization of the morphology and microstructure of the two samples was conducted using SEM and TEM. Approximately 57.5% of the total surface area is segmented into nano-sized regions by uniformly distributed TiO_2_ nanoparticles. A stable CEI formed on the surface of nano-modified flakes, endowing TO/GF with exceptional long-term cycling stability. Long-term cycling stability is a critical factor reflecting the electrochemical and structural stability of electrode systems. TO/GF exhibits remarkably high cycling stability, capable of enduring 10,000 stable discharge/charge cycles within a voltage window of 3.0–4.98 V at a current density of 5 C. In contrast, the cycling stability of GF is significantly inferior to that of TO/GF, with a drastic capacity drop after 1000 cycles and continuous capacity decay during cycling. The initial reversible capacity of TO/GF reaches 72.5 mA h g^−1^. Charge transfer resistance (Rct) and CEI resistance (R_CEI_) are robust indicators of the electrochemical reversibility and structural stability of high-voltage electrode systems. Thus, serial EIS measurements and data fitting were performed on both samples to reveal the variation of Rct and R_CEI_ with discharge/charge cycles. The R_CEI_ of GF rapidly increases with cycling, reaching 98 Ω after 1000 cycles. In contrast, the R_CEI_ of TO/GF increases to 38 Ω after the initial 100 cycles, with minimal subsequent changes, ultimately reaching 48 Ω after 10,000 cycles. R_CEI_ serves as a powerful indicator of the stability of the CEI formed on the electrode surface. The evolution of R_CEI_ for TO/GF indicates that the formed CEI is highly stable after 100 discharge/charge cycles, thus enabling the TO/GF electrode to maintain stability over 10,000 discharge/charge cycles. In contrast, it can be inferred that the CEI of GF thickens with increasing cycle numbers, fully illustrating that electrolyte decomposition is the primary cause of the rapid capacity decay of GF. XRD spectra reveal a significant amount of LiF in the GF electrode, consistent with EDS analysis results. However, no LiF reflection was detected in the TO/GF electrode. Calculations show that the (002) lattice spacing of graphite flakes in the GF and TO/GF electrodes is 0.3356 nm and 0.3353 nm, respectively. Consequently, the lattice of GF expands by 0.15% after cycling. However, TO/GF maintains zero lattice expansion of graphite flakes even after 10,000 cycles, demonstrating extremely high structural stability. Qin et al.^165^ achieved significant design advancements by successfully preparing carbon-coated hollow aluminum nanospheres (CHAA) on aluminum foil as the negative electrode for DIBs. The application of these hollow nanostructures on aluminum foil served multiple purposes, aiding in alleviating stress during aluminum-lithium (AlLi) alloying and reducing the energy barrier for alloy formation (Figure 12C). The research on the modification of graphite cathode surfaces with some coating materials to improve CEI performance is summarized in Table 2.

**Table 2:** Several surface coatings for improving the CEI and electrochemical performance

Surface coating materials	Cathode	Electrolyte	Voltage range (V)	Capacity (mAh g^−1^)	Year and ref.
Li_4_Ti_5_O_12_ (LTO)	MCMB+1.5 wt % LTO	1.0 M LiPF_6_ in EMC:SL (1:4 by volume)	5.4-3.0	86.9 (5 C)	Han et al., 2019^159^
TiO_2_	TO/GF	3.5 M LiPF_6_ in EMC	5.0–3.0	73.7 (2 C)	Han et al., 2021^164^
Al_2_O_3_	PG@Al_2_O_3_	4 M LiPF_6_ in EMC	5.0–3.0	72 (200 mA g^−1^)	Li et al., 2022^166^
Single-walled carbon nanotubes (SWCNTs)	GSC	4 M LiPF_6_ in EMC	5.0–3.0	100.5 (200 mA g^−1^)	Li et al., 2022^87^
Ascorbic acid	rGO	1 M LiPF_6_ in EC:EMC:DMC (1:1:1 by volume)	5.0–3.0	94 (1 A g^−1^)	Qi et al., 2021^167^
Sodium ascorbate	CFMS	0.8 M KPF_6_ in EC:DMC (1:1 by volume)	3.8-0.01	90 (2000 mA g^−1^)	Feng et al., 2019^168^
LiFePO_4_	MCMB@LFP	9.25 M LiTFSI in DMC	3.5-0.5	117 (0.2 A g^−1^)	Cheng et al., 2022^169^
Ni_3_S_2_	Ni_3_S_2_/graphene	AlCl_3_: [EMIm]Al_x_Cl_y_ (1.3:1 by weight)	2–0.1	60 (100 mA g^−1^)	Wang et al., 2016^170^
Pitch	Pitch-derived soft carbon	4 M LiTFSI in EC:DEC:DMC (1:1:1 by volume)	5.0–2.0	72 (100 mA g^−1^)	Ghosh et al., 2023^171^
Diethylenetria-minepenta (methylene-phosphonic acid) (DTPMP)	Graphite@DTPMP	4.0 M LiPF_6_ in EMC	5.0–3.0	88.4 (100 mA g^−1^)	Zhang et al., 2023^158^

To enhance the energy storage performance of lithium-graphite DIBs to practical levels, Li et al.^166^ adopted a dual-layer modification strategy, strengthening the electrolyte interfaces of both the negative and positive electrodes. On the negative electrode side, they constructed a three-dimensional carbon framework to achieve uniform Li flux, thus guiding stable SEI (on anode) formation, uniform Li deposition, inhibiting lithium dendrite growth, and mitigating problems caused by volume expansion (Figure 12D). On the positive electrode side, others coated a layer of rigid inert material on the graphite positive electrode surface to enhance CEI stability, thus maintaining the structural integrity of the graphite positive electrode (Figure 12E). Comparisons of their performance indicate that each electrode modification increases capacity retention during cycling. The compatibility between electrolytes and two electrodes, as well as the structure and stability between them, play crucial roles in determining low-temperature charge transfer.^88^ They proposed synergistic enhancement at the interfaces of positive and negative electrodes to improve the cycling stability and adaptability of DIB over a wide temperature range from 40°C to −25°C. Apart from severe electrolyte decomposition at high voltages, the significant volume expansion of graphite positive electrodes during repeated anion (de-)intercalation processes can lead to damage to the CEI and even delamination of the graphite layer. As a conductively poor but rigid intermediate phase, amorphous Al_2_O_3_ layer is coated on the graphite positive electrode (PG@Al_2_O_3_). In DIB, unilateral interfacial modification effectively suppresses side reactions at the electrode/electrolyte interface, reduces electrode polarization, and enhances CE at all temperatures. Typically, after 1000 cycles under conditions of 0°C and 1 A g^−1^, the capacity retention rate is 93%, whereas for DIB without unilateral interfacial protection under the same conditions, the capacity retention rate is only 50%. More importantly, at −25°C and 50 mA g^−1^ conditions, the reversible capacity of unilateral interfacially modified DIB increases to 42 mAh g^−1^. The composition of the CEI formed after cycling at different temperatures was investigated by XPS for bare graphite and PG@Al_2_O_3_ positive electrodes. Regardless of whether the Al_2_O_3_ layer was introduced, the amount of by-products formed after cycling at 0°C and −15°C was higher than that at 25°C and 40°C, indicating more severe electrolyte decomposition on the positive electrode side at low temperatures. When the number of cycles increased from 200 to 600, the variation in lithium content on the PG@Al_2_O_3_ positive electrode was much smaller than that on the bare graphite interface at all temperatures. This result suggests that the Al_2_O_3_ coating significantly improves the chemical stability of CEI during cycling. After 600 cycles, the interplanar spacing corresponding to the (002) plane of the PG@Al_2_O_3_ positive electrode is smaller compared to that of the bare graphite positive electrode, indicating the effective mitigation of graphite layer expansion due to the presence of a rigid Al_2_O_3_ coating layer on the graphite positive electrode. To investigate the evolution of the overall structure of graphite, XRD measurements were conducted on the cathode sides of pristine graphite (named PG-C) and the cathode sides of synergistic enhancement (named SE-C) after cycling 200 and 600 times at 25°C. After 200 cycles, there were no significant changes observed in PG-C and SE-C. However, after 600 cycles, the intensity of the (002) peak in PG-C decreased. Additionally, the peak of PG-C (002) shifted to a lower angle of 26.46° compared to the peak of SE-C (26.56°), indicating expansion of the graphite flakes after anion de/intercalation. Similar trends were observed in XRD spectra at other temperatures, with SE-C showing greater peak intensity and smaller peak shifts after cycling. It is noteworthy that electrodes cycled at low temperatures exhibited weaker peak intensities, suggesting more severe instability of the CEI due to anion de/intercalation in graphite at low temperatures. The thick and solid CEI formed at low temperatures could shield the diffraction of graphite, resulting in weaker diffraction intensity. The higher contents of F and Li after low-temperature cycling compared to room temperature and high-temperature cycling further confirmed this speculation. This innovative approach provides an effective pathway for the development of DIBs technology, achieving sustained high performance under different temperature conditions. Similarly, Li et al.^87^ conducted an integrated design by simultaneously optimizing the structures of the positive and negative electrodes. They created a self-supporting graphite-based flexible composite electrode (GSC) by combining single-walled carbon nanotubes (SWCNTs) with graphite (Figure 12F), which does not contain an aluminum current collector. This design effectively constructed a three-dimensional conductive network for the graphite positive electrode while forming a stable CEI layer on the surface of the graphite positive electrode. XRD was used to characterize the structures of graphite, single-walled carbon nanotubes, and GSC (Figure 12G). A reflection at 30.1° corresponding to the (002) plane, with a spacing of 26.5 nm, was consistent with standard card JCPDS#41–1487, exhibiting a typical two-dimensional layered structure. The XRD spectrum of single-walled carbon nanotubes showed a broad diffraction peak around 41°, resembling the characteristics of amorphous carbon. However, in the Raman spectroscopy characterization (Figure 12H), the spectrum of single-walled carbon nanotubes was highly consistent with that of graphite, but no D-band peak representing defects was observed, only the dominant G-band peak representing graphitic structure. This indicated higher quality and fewer defects in single-walled carbon nanotubes. The morphology of the graphite positive electrode (Figure 12I), composed of irregular particles with diameters ranging from 15 to 30 μm, while the single-walled carbon nanotubes appear as nanofibers with diameters ranging from 10 to 20 nm (Figure 12J). The SEM image of GSC revealed a small amount of scattered conductive carbon black particles on the surface of GSC (Figure 12K). These characterization results collectively depicted the structural characteristics of graphite, single-walled carbon nanotubes, and GSC, providing important information for further understanding their performance in applications such as batteries.

In summary, by optimizing through element doping, artificial CEI and structure design, the performance of DIBs can be significantly improved. These methods can enhance the energy density, cycle stability, and power output of batteries, providing important avenues for achieving reliable and efficient DIB technology.

## Advanced characterizations and analysis methods

In order to gain deeper insights into the electrochemical reaction processes at the electrode interface, advanced characterization and testing methods are required. Due to the thin thickness of CEI and its sensitivity to environmental factors such as oxygen and humidity, the study process is highly complex. Therefore, there is a great need to adopt advanced characterization techniques, electrochemical analysis methods, and theoretical calculations to investigate CEI. Through the application of these methods, improvements and optimizations in battery technology can be facilitated.

**Figure 13:**
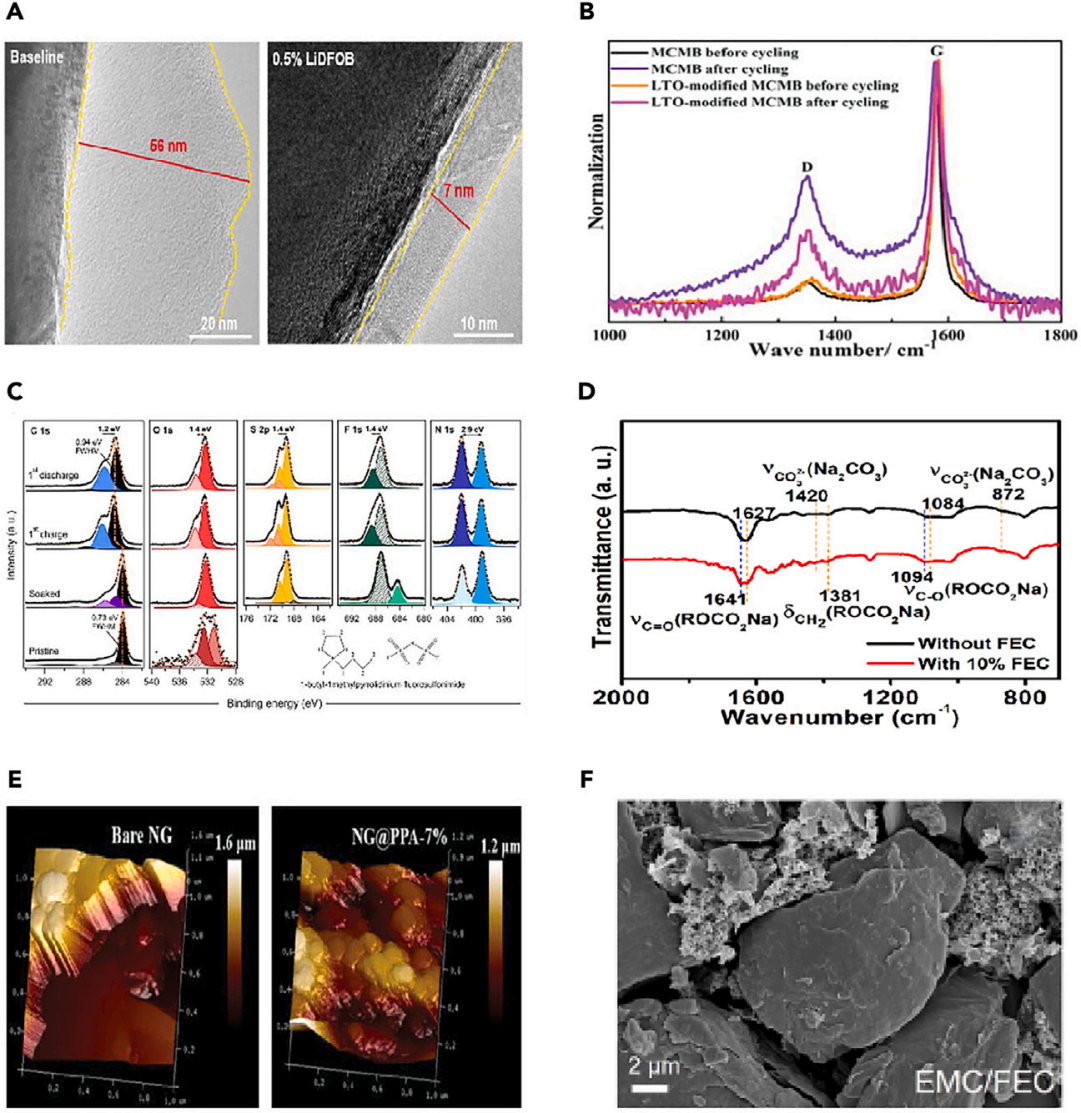
The list of instruments for the characterization of CEI (A) TEM. Reprinted with permission from Wang et al.^48^ Copyright 2021 Wiley-VCH GmbH. (B) RAM. Reprinted with permission from Han et al.^159^ Copyright 2019 WILEY-VCH Verlag GmbH & Co. KGaA, Weinheim. (C) XPS. Reprinted with permission from Kotronia et al.^116^ Copyright 2021 American Chemical Society. (D) FTIR. Reprinted with permission from Yu et al.^27^ Copyright 2021 American Chemical Society. (E) AFM. Reprinted with permission from Zhang et al.^83^ Copyright 2022 Wiley - VCH GmbH. (F) SEM. Reprinted with permission from He et al.^131^ Copyright 2023 American Chemical Society.

Characterization and analysis methods of the CEI layer in DIBs are continuously evolving. Here are some advanced methods: 1) TEM: TEM technology can observe the formation and evolution process of the CEI layer. Information about the composition, morphology, and crystalline properties of the CEI layer can be obtained through TEM images and electron diffraction (Figure 13A).^48^ 2) Raman spectroscopy: Raman spectroscopy provides information about the chemical composition and structure of the CEI layer. By observing and analyzing the peak positions and intensities of Raman spectra, the formation and breakage processes of chemical bonds in the CEI layer can be understood (Figure 13B).^159^ 3) XPS: XPS is used to characterize the surface composition and chemical state of the CEI layer. Analysis of XPS spectra can provide information about the oxidation states of elements, chemical bonds, and oxygen-containing functional groups in the CEI layer (Figure 13C).^116^ 4) FT-IR: Infrared spectroscopy is used to analyze functional groups and chemical bonds in the CEI layer. By observing and analyzing the absorption peaks of infrared spectra, the chemical composition and structure of the CEI layer can also be understood (Figure 13D).^27^ 5) Atomic Force Microscopy (AFM): AFM allows high-resolution observation and measurement of the surface morphology and roughness of the CEI layer. AFM images provide nanoscale morphology information of the CEI layer (Figure 13E).^83^ 6) SEM: SEM plays a crucial role in the study of the graphite surface CEI layer. Through SEM, people can visually observe the microstructure of the graphite surface, inspect the morphology, and defects of the surface layer (Figure 13F).^131^ SEM with EDS also provides the capability for compositional analysis, revealing the chemical composition of the CEI layer, thereby providing important information for battery material research. As technology further advances, more new methods will emerge to address the characterization and analysis of CEI layers.

## Summary and outlook

This review introduces the latest progress in stabilizing the CEI on graphite cathode in DIBs, encompassing strategies such as the introduction of additives and binders, the improvement of electrolyte composition, artificial CEI, graphite surface modification, and coatings. These approaches aim to enhance the stability of the CEI. Although the aforementioned methods have demonstrated promising results, a series of challenges still remain to be addressed. Particularly, under high voltage conditions, the reaction between the cathode and the electrolyte may intensify, leading to the thickening and instability of the CEI. An ideal CEI should possess key properties such as high ionic conductivity, low electronic conductivity, mechanical stability, and high chemical stability. To achieve high energy density and excellent cycling performance in DIBs, further research on the formation and evolution of the CEI is necessary. Research in this field will provide important insights for advancing the future development of battery technology.

In future research, particular emphasis should be placed on the following key aspects: Future research directions include 1) deepening the understanding of the formation mechanisms of the CEI layer, especially under the influence of anion transport mechanisms in DIBs. This requires a more comprehensive exploration of the kinetics and thermodynamics of CEI formation to more accurately control its structure and properties. 2) The developments of more precise and efficient CEI layer evaluation techniques are crucial. These evaluation techniques will help accurately understand the chemical and electrochemical properties of the CEI layer, providing more targeted guidance for optimization strategies. The graphite cathode in DIB tends to have strong stability, which has an advantage over other batteries. 3) Exploring new materials and technologies is also a focus of future research. 4) The future development of graphite cathode CEI modification in DIBs should include the construction of suitable anion-transporting CEI on the graphite cathode surface. Compared to conventional cation batteries, DIBs employ two types of ions for charge storage and transport. Typically, one cation and one anion participate in electrochemical reactions in the electrolyte. During charging, the anion is intercalated in the positive electrode and the cation in the negative electrode. Therefore, in order to increase the power density of DIBs, it is necessary to construct a CEI suitable for anion transport on the surface of the positive electrode because the capacity and cycling performance of the battery can be substantially increased by efficiently and usefully intercalating the anion in the graphite layer without destroying the structure and dimensions of the graphite layer during the intercalating process. 5) In addition, the construction of chemically stable high-voltage CEI is also an important development direction. Although high-voltage cycling can improve the energy density of DIBs, it may also lead to the decomposition of the electrolyte and the distortion and defects of the graphite layer, which may affect the cycling performance and safety of the battery. The introduction of new additives, the use of nanomaterials, or the adoption of coating techniques are expected to enhance the stability and performance of the CEI layer, thereby improving the overall performance of the DIBs. It is believed that through continuous in-depth research and innovation, the performance and stability of the CEI layer in graphite positive electrodes of DIBs will be improved, bringing about significant progress and contributions to energy storage technology development.
